# Stress‐Induced Reorganization of Proteasomes Through Diffusion and Cytoskeleton‐Dependent Mechanisms

**DOI:** 10.1002/smll.202506260

**Published:** 2025-11-24

**Authors:** Michael J Morten, Yu Zhang, Bing Li, Jonathan X Meng, Kun Jiang, Liina Sirvio, Ji‐Eun Lee, Anna H Lippert, Matilda Burridge, Katiuska Daniela Pulgar Prieto, Alexander R Carr, Aleks Ponjavic, Steven F Lee, Daniel Finley, David Klenerman, Yu Ye

**Affiliations:** ^1^ Department of Brain Sciences Imperial College London 86 Wood Lane London W12 0BZ UK; ^2^ Department of Chemistry University of Cambridge Lensfield Road Cambridge CB2 1EW UK; ^3^ Department of Cell Biology Harvard Medical School Longwood Avenue Boston MA 02115 USA; ^4^ Present address: Institute for Systems Immunology Julius‐Maximilians University Würzburg Versbacher Strasse 97078 Würzburg Germany; ^5^ Present address: School of Physics and Astronomy University of Leeds LS2 9JT Leeds United Kingdom; ^6^ Present address: School of Nutrition and Food Science University of Leeds LS2 9JT Leeds United Kingdom

**Keywords:** nanopipette injection, protein aggregation and degradation, single‐cell patch clamp, super‐resolution and light‐sheet microscopy, ubiquitin‐proteasome system

## Abstract

Proteasomes are abundant molecular machines distributed throughout the eukaryotic cell to facilitate protein degradation. How their dynamic localization adapts to proteostasis requirements remains an area of active study. Recent studies from the authors show that proteotoxic stress induced by alpha‐synuclein aggregates triggers proteasome reorganization into foci bodies, termed transient aggregate‐associated droplets (TAADs). Here, advanced imaging and biophysical techniques are combined to examine proteasome reorganization and TAAD formation. Using single‐molecule localization and light‐sheet microscopy, redistribution of subcellular proteasome density is quantified in response to alpha‐synuclein aggregates. Interestingly, the ratio of 20S proteasome core particles capped by 19S regulatory particles (∼60%) remains constant during proteotoxic stress. Delivery of aggregates by nanopipette injection reveals that TAAD formation is cytoskeleton‐dependent, suggesting that directed transport is required for proteasome reorganization. Single‐cell patch clamp further shows that cytoskeleton‐dependent proteasome movement is linked to cell depolarization, implying that membrane potential can directly modulate proteasome localization. Single‐particle tracking analysis detects the presence of both rapid‐ and slow‐moving proteasome populations with proteotoxic stress shifting their motion towards confined diffusion within TAADs. Together, these results demonstrate that proteasomes adopt distinct modes of motion depending on cellular requirements and become restricted upon aggregate invasion, highlighting a tightly regulated system of proteasome organization for selective proteostasis during stress.

## Graphical abstract

Proteotoxic stress induced by alpha‐synuclein aggregates drives rapid, cytoskeleton‐dependent re‐localization of mammalian proteasomes and formation of transient aggregate‐associated droplets (TAADs). Single‐molecule localization microscopy, light‐sheet imaging, nanopipette injection, single‐cell patch clamp, and single‐particle tracking reveal a constant ratio of proteasome core to regulatory particles but a stress‐dependent shift from global diffusion to confined motion and altered subcellular density, tightly regulating proteasome availability for selective proteostasis.

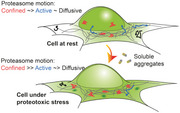

## 1 Introduction

The ubiquitin‐proteasome system controls the level of cellular proteins by selective degradation and thereby regulates key signaling pathway, such as inflammation, oxidative stress and the, unfolded protein response, as well as clearance of protein aggregates.^[^ ^1^, ^2^, ^3^, ^4^, ^5^ ^]^ Ubiquitin‐dependent degradation is canonically carried out by the proteasome holoenzyme, which is assembled from 20S core particles (CP) and 19S regulatory particles (RP).^[^ ^6^ ^]^ The holoenzyme is believed to be the preferred proteasome assembly and may reversibly disassemble into CPs and RPs in the cell^[^ ^7^ ^]^ (Figure S1A, Supporting Information). On their own, CPs are thought to degrade unfolded and disordered proteins, including nascent protein turnover during neuronal stimulation.^[^ ^8^ ^]^ Free RPs have been found with increased abundance at the synapse and are suggested to regulate synaptic transmission.^[^ ^9^ ^]^ Substrates selected for degradation are normally modified by ubiquitin moieties, which facilitate recognition and processing at the proteasome.^[^ ^10^ ^]^ A recent report has added to this view and demonstrated further selective substrate degradation in a ubiquitin‐independent manner by the holoenzyme,^[^ ^11^ ^]^ underscoring the versatile functions and regulatory roles assigned to the proteasome system.

Early studies have reported that proteasomes are present throughout the cell,^[^ ^12^, ^13^, ^14^ ^]^ presumably to facilitate the local protein turnover, though the observed relative distribution can vary between biological conditions and imaging methods.^[^ ^15^, ^16^, ^17^ ^]^ Altering physiological conditions such as hypertonic stress, osmotic shock, and starvation has been shown to drive proteasomes into foci bodies in a liquid–liquid phase separation (LLPS)‐dependent manner.^[^ ^18^ ^]^ These LLPS‐foci have been given multiple names, including clastosomes, senescence‐associated nuclear proteasome foci, and proteasome storage granules (PSGs).^[^ ^19^ ^]^ Their formations are thought to help rebalance proteostasis in response to the various types of cell stress; PSGs are associated with a protective role to sequester proteasomes during starvation,^[^ ^19^ ^]^ and clastosomes may function as degradation centers in response to hypertonic stress.^[^ ^18^ ^]^ More recently, we postulated that certain foci may serve to concentrate proteasomes with their co‐proteins and enzymes of the ubiquitin system to facilitate disaggregation and degradation. We termed such foci transient aggregate‐associated droplets (TAADs)^[^ ^2^ ^]^ and demonstrated that TAADs have molecular properties distinct from LLPS‐foci in an accompanying story.^[^ ^20^ ^]^ The formation of these TAADs required reorganization of cellular proteasomes, however the cellular mechanisms enabling their formation are unknown.

In this work, we adapted several advanced imaging and analysis techniques to study the reorganization of proteasomes in response to toxic alpha‐synuclein (aS) aggregates that have entered the cell. Quantifying the subcellular localization and the ratio of the different proteasome particles by single‐molecule localization microscopy (SMLM) revealed stress‐induced changes in their cellular distribution but not in their ratio during TAAD formation. We subsequently showed that TAAD formation was cytoskeleton‐dependent by nanopipette aggregate injection. We could further link cytoskeleton‐dependent transport of proteasomes to changes in membrane potential and used single‐cell patch clamp to show that hyper‐ and depolarization also relocalized proteasomes. Critically, using single‐particle tracking (SPT) analysis approaches, we determined that TAAD formation was accompanied by a global reduction in freely moving proteasomes and an increase in confined proteasomes. Together, our data suggest that proteasomes are dynamic particles that change their mode of motion, cellular distribution and local concentration in response to external stimuli as required by the cell.

## 2 Results

### 2.1 Proteotoxic Stress Induces Re‐Organization of Proteasomes

**Figure 1:**
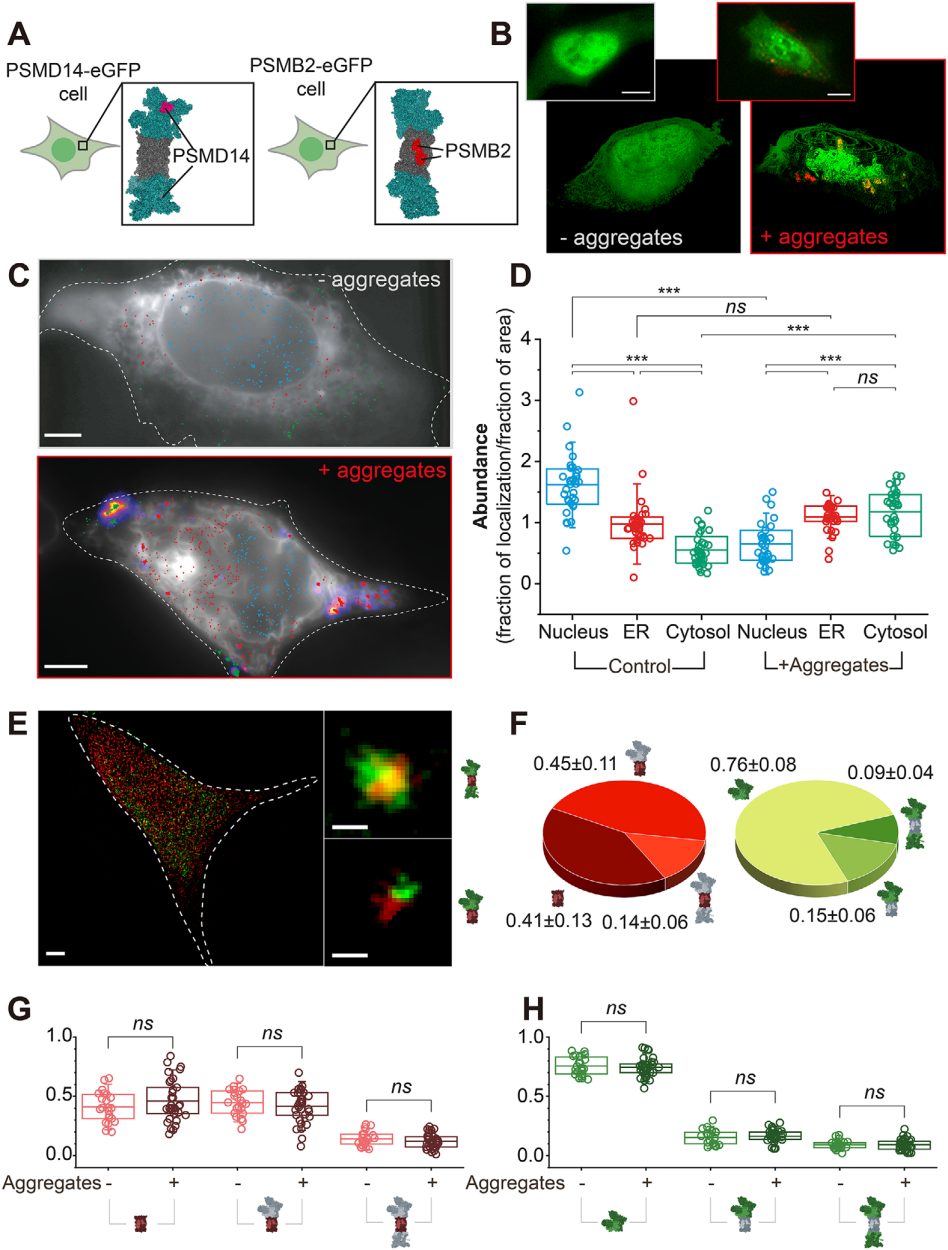
Proteotoxic stress induces major redistribution of proteasome particles. A) Cartoon representation of CRISPR‐edited cells expressing subunit of the RP (PSMD14, *left*) or CP (PSMB2, *right*) in tandem with FP at the C‐terminus. B) A typical PSMD14‐eGFP cell reconstructed in 3D from HILO imaging with 100 nm step size z‐stack before (*left*) and 24 hr after (*right*) incubation with aS aggregates (Alexa647 partial labeling). The inset shows a 2D image with a scale bar = 5 µm. C) HILO imaging of individual proteasomal particles in PSMD14‐mEos cells expressing an ER marker, eGFP‐Sec61 (white), at rest versus after 24 hrs incubation with aS‐Alexa647 (fire‐LUT). Cells were imaged using SMLM (see Experimental Section). Reconstructed RPs localized to the nucleus (blue), overlapping with the ER (red), or the cytosol (green) are color‐coded. Scale bars = 1 µm. D) Calculated relative abundance of PSMD14‐mEos found in each subcellular compartment before and after aS aggregate incubation presented in box‐plots (*n* = 35 and *n* = 31 cells, respectively). E) Immunofluorescence labeling and SMLM imaging of a typical HEK293A cell for CP (red) and RP (green) enabled calculation of capped versus uncapped proteasome CP ratios. Scale bars represent 5 µm and 100 nm (inset). Inset with cartoon representations of singly‐ and doubly capped holoenzymes, are shown (*right*). F) Relative ratios of free CP and RP versus singly‐ and doubly capped holoenzymes in HEK293A are shown in pie charts. Mean ± standard deviation from 22 cells are presented to two decimal places. G–H) HEK293A cells were incubated with aS aggregates for 24 hrs (*n* = 33 cells) and labeled as in E, for fractional quantification of (G) CP and (H) RP. Box plots show that incubation of aggregates does not induce any significant changes between the relative ratios of free particles and holoenzymes. This is in line with observations made in differentiated SH‐SY5Y neuroblastoma cells (Figure S6, Supporting Information). Statistical significance was calculated using Student's *t*‐test, where no significant difference (n.s.) *p* > 0.05, ^*^ *p* < 0.05, ^**^ *p* < 0.01, and ^***^ *p* < 0.001.

To study proteasome dynamics in cells, we used CRISPR‐Cas9 to edit the genomic sequences of PSMD14/RPN11 or PSMB2/PRE1, subunits of the RP or CP, respectively, to be expressed in tandem with fluorescent protein (FP) sequences encoding eGFP or mEos (**Figure** **1**A; Figure S1A,B, Supporting Information, see Experimental Section). Monoclonal HEK293A cell lines expressing each of the four constructs (PSMD14‐eGFP, PSMB2‐eGFP, PSMD14‐mEos, or PSMB2‐mEos) were confirmed by genotyping, and incorporation of FP‐tagged subunits into proteasomal particles was verified by native gels (Figure S1C,D, Supporting Information), in line with previous studies.^[^ ^21^, ^22^, ^23^ ^]^ The ratios of FP‐tagged to untagged proteasomes were determined by densitometry analysis from Western blots (Figure S2, Supporting Information).

We examined the spatial distribution of proteasomes using total‐internal reflection fluorescence (TIRF) microscopy in highly inclined and laminated optical‐sheet (HILO)^[^ ^24^ ^]^ mode and by light‐sheet microscopy, confirming that both CP and RP were present throughout the cell, with strong presence in the nucleus (Figure 1B *left*; Figure S3A *left*, and Figure S3B *top*, Supporting Information). Significant proteasomal redistribution was observed upon proteotoxic stress, induced through incubation with pre‐assembled recombinant alpha‐synuclein (aS) aggregates that were partially labeled with Alexa Fluor 647, or Alexa647 (Figure 1B *right*; Figure S3A *right*, and Figure S3B *bottom*, Supporting Information). In addition to the global proteasomal redistribution, we also observed high fluorescence intensities from proteasomes in close proximity to the internalized aggregates, consistent with our proposed description of TAADs.^[^ ^2^, ^20^ ^]^ To quantify this global reorganization, we imaged PSMD14‐mEos or PSMB2‐mEos cells by SMLM and counted the number of proteasomes found either within the nucleus, co‐localizing with the ER, or residing elsewhere in the cytosol (see Experimental Section). Cells were fixed and imaged under HILO conditions, followed by SMLM reconstruction of individual PSMD14‐mEos localizations at high precision (≈4 nm)^[^ ^1^ ^]^ as shown in Figure S3C (Supporting Information). In resting cells, both CPs and RPs were densest in the nucleus and gradually reduced in density at the ER and in the cytosol (Figure 1C,D). This density gradient was shifted by aggregate‐induced proteotoxic stress, which caused significantly increased proteasome levels in the cytosol at the expense of reduced levels in the nucleus. We interpreted the two observed distributions representing a resting and a stressed state, where cells responded to invading aggregates by reorganizing proteasomes across the subcellular compartments. To rule out the possibility that apoptotic processes were responsible for the shift in proteasomal distribution, we immunostained for Annexin V in resting or aggregate‐stressed cells and found no significant difference in proteasome distribution (Figure S4, Supporting Information). These results suggest that the shift in proteasomal distribution is a response to proteotoxic stress rather than programmed cell death.

We subsequently assembled hypertonic‐induced LLPS‐foci by adding CaCl_2_^[^ ^18^, ^25^ ^]^ to the cell media (Figure S5A,B, Supporting Information) and compared these to TAADs induced by aS aggregates. LLPS‐foci were distinct from aggregate‐induced TAADs and were smaller, rounder, more numerous, and mostly localized to the nucleus (inset Figure S5B, Supporting Information). This is consistent with an accompanying study from our group describing the molecular properties and functions of TAADs,^[^ ^20^ ^]^ which suggests that proteotoxic and hypertonic stress induce distinct types of proteasome formations specific to the type of stress signal.

To examine if proteasome assemblies were altered by stress, we quantified the level of free CP and RP versus singly‐ or doubly‐capped proteasome holoenzymes using SMLM (Figure 1E and Experimental Section). In resting cells, we observed high correlation between CPs and RPs, with the majority of CP associated with one (0.45 ± 0.11) or two (0.14 ± 0.06) RP and therefore forming singly‐ or doubly‐capped holoenzymes, respectively (Figure 1F, *left*). A significant fraction (0.41 ± 0.13) of CP was detected without association with RP and therefore classified as free particles. Repeating the relative quantification for RPs, we observed similar levels of singly‐ (0.15 ± 0.06) and doubly‐capped (0.09 ± 0.04) holoenzymes, while most RPs (0.76 ± 0.07) remained as free particles (Figure 1F, *right*). The observed differences in relative levels of free CP and free RP ratios are consistent with a recent observation in neurons,^[^ ^9^ ^]^ and suggest that the majority of CP activities are coupled to those of the RP, while free RPs themselves may have distinct functions, for instance, as deubiquitinases^[^ ^9^ ^]^ or chaperones of misfolded proteins.^[^ ^26^ ^]^

These observations were confirmed in differentiated SH‐SY5Y neuroblastoma cells (Figure S6A,B, Supporting Information), with similar distributions of CPs (0.32 ± 0.18, 0.22 ± 0.19, 0.46 ± 0.15 for singly‐, doubly‐capped holoenzymes and free particles, respectively), whereas RPs were found with a somewhat decreased fraction of free particles (0.25 ± 0.16, 0.31 ± 0.26, 0.45 ± 0.20). Intriguingly, proteotoxic stress by aS aggregates did not quantitatively change the relative ratios of free CPs and RPs in either HEK293A or SH‐SY5Y cells (Figure 1G,H; Figure S6C,D, Supporting Information). Our observations together indicate that while proteasomes are dynamically distributed, the ratio between free particles and assembled holoenzymes is largely maintained, which potentially carries significance for proteostasis in stressed cells.

### 2.2 Assembly of TAADs is Dependent on the Cytoskeleton

**Figure 2:**
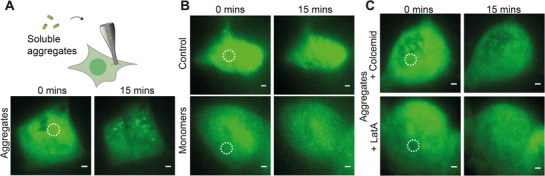
Proteasome response to aggregates in the cell is cytoskeleton‐dependent. A) Schematic representation of aS aggregate injection into HEK293A cells expressing PSMD14‐eGFP using a nanopipette (*top*) and HILO images showing proteasomes forming foci in response to aS aggregates (*bottom*, see also Video S1, Supporting Information and Experimental Section). B) Cells injected with buffer (*top*) and aS monomers (*bottom*, and Video S2, Supporting Information) did not induce foci formation. C) No foci were observed in cells treated with 5 µm Colcemid (*top*) or Latrunculin A (LatA) (*bottom*) before injecting aggregates (see also Videos S3 and S4, Supporting Information), which disrupt the microtubule and actin networks, respectively. The injection positions are marked by circles with dotted lines. All images show representative results from at least three biological repeats.

While LLPS‐foci emerge almost instantaneously after hypertonic stress, the formation of TAADs spans over several hours.^[^ ^20^ ^]^ We therefore accelerated the internalization of aS aggregates using a nanopipette for injection into PSMD14‐eGFP cells. Nanopipette injection is a bespoke approach enabling delivery of soluble aggregates at precise positions in the cytosol of a live cell,^[^ ^27^ ^]^ which would enable us to also directly validate the relationship between proteotoxicity and TAAD formation. Aggregates released through the nanopipette into the cytosol induced TAAD formation within minutes (**Figure** **2**A), while no response was observed when a buffer control or aS monomers at the equivalent concentration were injected (Figure 2B). We therefore attribute the formation of TAADs to aggregate induction, rather than the presence of their constituent aS proteins.

We subsequently tested if TAAD formation was driven by active transport through the cytoskeleton or facilitated through diffusion. Aggregate injection was repeated in PSMD14‐eGFP cells pre‐treated for 10 min with Colcemid or Latrunculin A (LatA), which depolymerized microtubules or actin filaments, respectively (Figure 2C). No TAAD formation was observed under either condition, suggesting that cytoskeletal integrity was required to concentrate proteasomes into TAADs. As validation, we labeled the microtubule network using SiR‐tubulin, which allowed us to determine whether microtubules were depolymerized in the specific cell, and showed that TAADs were only observed in cells with intact cytoskeleton (Figure S7, Supporting Information).

Since LLPS‐foci were primarily localized in the nucleus, we thought their formation may be independent of the cytoskeleton. To test this, the cytoskeleton in PSMD14‐eGFP and PSMB2‐eGFP cells was depolymerized with Colcemid or LatA prior to hypertonic stress. LLPS‐foci appeared at similar levels to those observed in cells with intact cytoskeletal integrity, and remained present in the nucleus after 10 min (see Figure S5C,D, Supporting Information), indicating that hypertonic stress‐induced proteasome response is independent of the integrity of the cytoskeleton. Together, our results suggest that LLPS‐foci and TAADs differ in their mode of formation, and cells may employ distinct mechanisms to reorganize proteasomes in response to the type of stress.

### 2.3 Cytoskeleton‐Dependent Proteasomal Movement is Linked to Membrane Potential

**Figure 3:**
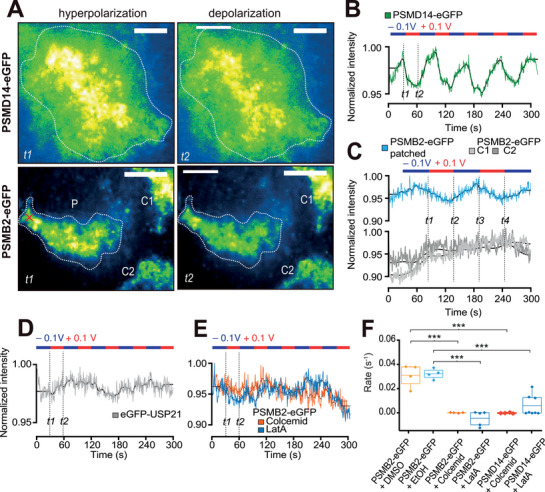
Membrane potential modulates proteasome localization. A) Unpatched and patched PSMD14‐eGFP (top) and PSMB2‐eGFP (bottom) cells are imaged in TIRF mode with snapshots shown from various time points (four panels of *t1‐t2* as in B and C), where the normalized fluorescence intensity is colored (see Experimental Section for image processing and bleach correction). Three PSMB2‐eGFP cells are visible in the bottom field of view, with only the cell on the left under patch clamp. The patch clamp position is marked with an “×” (red) on the cell (*P*) in panel *t1*. Regions of the patched cells used for intensity plots are marked in panel *t1* as dotted lines. Intensity changes in control cells *C1* and *C2* are shown in C. See also Video S5 (Supporting Information). B) Changes in the normalized fluorescence signal are shown over time for the PSMD14‐eGFP cell in A. Solid black lines represent signals after applying a filter in the frequency dimension to remove modulations smaller than the oscillation in potential. The clamped potentials applied are indicated on the top and oscillated between +100 mV (red) and −100 mV (blue) every 30 s (see also Video S6, Supporting Information). C) Changes in normalized fluorescence signal over time in PSMB2‐eGFP cells are shown for the patched cell and two control cells shown in A. D) Cells expressing eGFP‐USP21 did not show any change in fluorescence intensity following changes in membrane potential induced by a patch clamp (see also Video S7, Supporting Information). E) Depolymerization of microtubules and actin filaments affects voltage‐induced proteasome re‐localization. PSMB2‐eGFP cells were treated with 5 µm Colcemid or Latrunculin A (LatA) for 20 min at 37 °C and washed with cell media before mounted for TIRF imaging. Fluorescence intensity traces of the cells are plotted over time, and the applied potential at each time indicated. PSMB2‐eGFP cells treated with DMSO or ethanol (EtOH) vehicle controls showed similar results (see Video S8, Supporting Information). F) Repeated measurements of patch clamp applied on PSMB2‐eGFP cells treated with (*from left*) DMSO (mean rate with SD = 0.031 ± 0.009 nFI s^−1^, *n* = 4), EtOH (0.032 ± 0.004 nFI s^−1^, *n* = 4), Colcemid (0.0001 ± 0.0003 nFI s^−1^, *n* = 4), LatA (−0.004 ± 0.006 nFI s^−1^, *n* = 5). PSMD14‐eGFP cells were also treated with Colcemid (−0.0002 ± 0.0002 nFI s^−1^, *n* = 4) and LatA (0.006 ± 0.009 nFI s^−1^, *n* = 8). All images are contrast‐adjusted for optimal representation of the difference in intensity between hyper‐ and depolarized states.

Invading aS aggregates are thought to interact with and penetrate the plasma membrane, which causes cell depolarization,^[^ ^28^ ^]^ a phenomenon that has in turn been linked to diverting proteasome movements.^[^ ^29^ ^]^ We therefore tested whether membrane potential (V_m_) could orchestrate proteasome transport in PSMD14‐eGFP cells, which may explain the observed TAAD formation. Single‐cell patch clamp experiments were performed to determine the resting potential of PSMB2‐eGFP cells (Figure S8A,B, Supporting Information), followed by measuring the current response under depolarizing and hyperpolarizing potentials (Figure S8C,D, Supporting Information). Combining single‐cell patch clamp with TIRF microscopy, we found that hyperpolarization induced increased fluorescence intensity within the evanescent field (**Figure** **3**A–C). Because the evanescent field of TIRF preferentially illuminates fluorophores closest to the coverslip rather than the interior of the cell, our observation represents the accumulation of proteasomes to the cell periphery. Expectedly, depolarization had the opposite effect and decreased the detected fluorescence intensity as proteasomes translocated into the cell interior. Oscillations in fluorescence intensity with voltage can be detected across the clamped cell, while the unclamped neighboring cells were unaffected (C1 and C2 cells, Figure 3A,C). We did not notice morphological changes of the cell during patch clamp under our experimental conditions, and a control protein of the ubiquitin system, eGFP‐USP21,^[^ ^30^ ^]^ did not respond to changes in V_m_ under the same experimental conditions (Figure 3D; Figure S8E, Supporting Information). This suggests that the observed proteasome translocation is specifically related to membrane potential, toggling proteasomes toward the cell membrane or interior through hyper‐ or depolarization, respectively.

To test whether such voltage‐induced proteasome translocation is also cytoskeleton‐dependent, we performed patch clamp on cells with compromised cytoskeletons. PSMB2‐eGFP cells treated with Colcemid or LatA showed no proteasome translocation in response to hyper‐ or depolarization, while treatment with vehicle control (DMSO or EtOH) alone did not abolish voltage‐induced proteasome translocations (Figure 3E,F). Similar results were seen in PSMD14‐eGFP cells treated with Colcemid or LatA (Figure 3F), indicating that proteasome re‐organization is facilitated through cytoskeleton‐dependent transport and consistent with observations in Figure 2C. Possibly, such active transport would, upon aggregate internalization and depolarization, enable orchestrated relocalization of proteasomes toward the cell interior and act against their density gradient as shown in Figure 1C,D. Ultimately, this reorganization would rapidly concentrate and strengthen regional proteasomal activity targeting aggregates.

### 2.4 Two Distinct States of Proteasome Motions are Found in Resting and Stressed Cells

**Figure 4:**
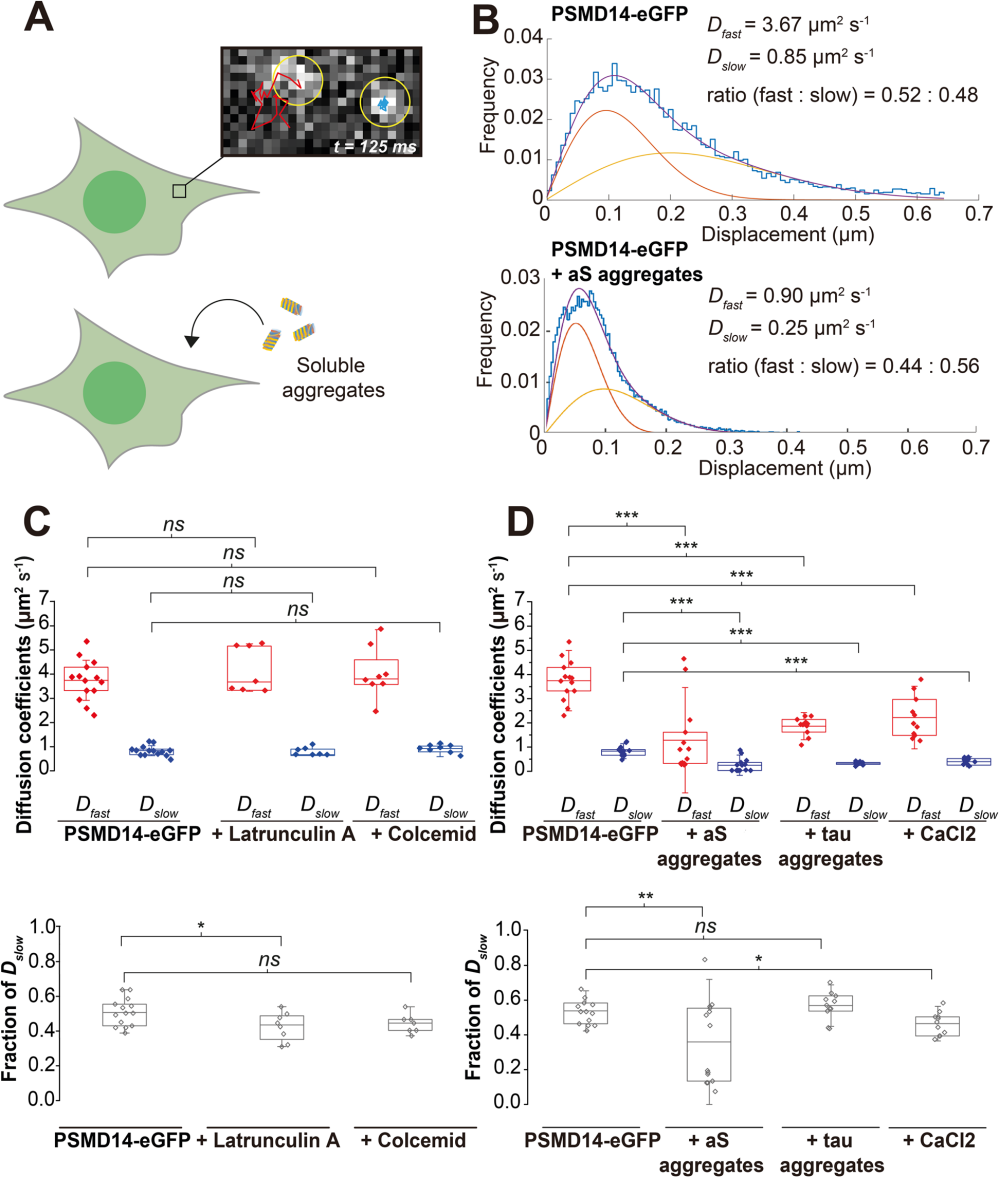
Single‐proteasome tracking (SPT) by TIRF imaging in live cells shows two distinct proteasome behaviors. A) A schematic of a proteasome moving fast (red tracks) inside a resting cell (*top*) expressing PSMD14‐eGFP (pixel size = 107 nm, see also Video S9, Supporting Information) and inside a cell incubated with aS aggregates (*bottom*). B) A representative jump distance (JD) analysis (frequency of displacement distances in cyan bars) of all PSMD14‐eGFP diffusion tracks detected within a cell (see also Video S10, Supporting Information). Diffusion coefficients for the fast‐ (*D_fast_*, yellow line), the slow population (*D_slow_*, red line), and overall fitting (purple line) are shown with the ratio of *D_fast_* to *D_slow_* for this particular cell at resting state (*top*). JD distribution shows a shift in the population distribution of the *D_fast_* and *D_slow_* proteasomes in response to aS aggregates (*bottom*). C) Box plots quantifying the *D_fast_* and *D_slow_* (*top*) and the fraction of proteasomes in the fast population (*bottom*) from individual cells treated with Colcemid (*n* = 8) or LatA (*n* = 7) compared with control cells treated with DMSO (*n* = 14). D) Similar box‐plots are shown describing cells at resting state (*n* = 12) and cells treated with aS aggregates (*n* = 14), tau aggregates (*n* = 11), and CaCl_2_ (*n* = 11).

Since the bulk of proteasome motion is thought to be achieved by diffusional movement,^[^ ^31^ ^]^ we examined their dynamics at a high time resolution by quantifying the movement of individual particles in resting or stressed cells using SPT. Our experimental setup and analytical methods for SPT have been described previously,^[^ ^32^ ^]^ and the localization precision was calculated as 25 ± 10 nm. Tracking was imaged in TIRF mode, and a statistical analysis by jump distances (JD) from individual tracks revealed two diffusion coefficients (*D_fast_ and D_slow_*) (see Experimental Section). Fitting the JD frequency distribution to models describing three or four diffusion coefficients did not improve the model fitting, confirming that the model is best described by two diffusional populations (Figure S9A,B, Supporting Information). Given that the ratio (1:1) and the diffusion coefficients (*D_fast_* at 3‐4 µm^2^ s^−1^; *D_slow_* ≈0.5 µm^2^ s^−1^) of both populations were similarly found in PSMD14‐eGFP (**Figure** **4**A,B) and PSMB2‐eGFP (Figure S9C,D, Supporting Information) cells at resting state, we attribute this as evidence that common transport mechanisms are acting on holoenzymes as on the free RPs and CPs.

Considering that TIRF imaging primarily detects proteasomes at the cell periphery within the evanescent field, we also validated diffusion coefficients and population ratios by repeating SPT in PSMB2‐mEos or PSMD14‐mEos cells in HILO mode^[^ ^24^ ^]^ to image the cell interior (Figure S9C,D, Supporting Information), achieving similar results as in Figure 4B. As a control, we performed SPT on eGFP‐USP21,^[^ ^33^ ^]^ an enzyme of the ubiquitin system similar in size to the free PSMD14 or PSMB2 but ≈40‐fold smaller than the proteasome holoenzyme, and ≈16‐ and 12‐fold smaller than the RP and CP, respectively. Given that the diffusion coefficients of eGFP‐USP21 were significantly higher (Figure S10, Supporting Information), we concluded that the eGFP‐ and mEos‐tagged PSMB2 and PSMD14 had been incorporated into respective particles, in agreement with Figure S1B (Supporting Information), and that our observation represented proteasome movements.

The presence of two diffusional populations is consistent with published works on yeast proteasomes, which assigned the slow population to interactions with macromolecular structures.^[^ ^31^ ^]^ We therefore examined if the slow‐moving population, represented by *D_slow_*, may be due to interactions with the cytoskeleton during the resting state. Surprisingly, treating PSMD14‐eGFP cells with Colcemid or LatA for 10 min prior to SPT did not alter *D_slow_* or *D_fast_*, nor did their population ratio change (Figure 4C), implying that alternative interactions may account for the slow‐moving proteasomes. We then tested how aggregate‐induced stress used to induce TAADs impacted *D_slow_* or *D_fast_* and again performed SPT on PSMD14‐eGFP cells with aS aggregates. Following overnight aggregate incubation to reach steady state, a significantly decreased *D_fast_* was detected, in line with reduced movements from proteasomes (Figure 4D). This was further reflected by a slightly increased ratio of the slow‐moving population. We attributed this population as the limited diffusion of proteasomes throughout the cell, some of which were now trapped in TAAD formations. In support of this, proteasomes in hypertonic‐stressed cells also produced a similar decrease in diffusion coefficients, albeit with a less drastic reduction in the magnitude of *D_fast_* (Figure 4D). Together, our findings point to the possibility that a fraction of proteasomes may be kept slow‐moving, which may enhance interaction with other large macromolecules in cells, such as with aggregates.

### 2.5 Proteasome Diffusion becomes more Confined upon Aggregate Internalization

**Figure 5:**
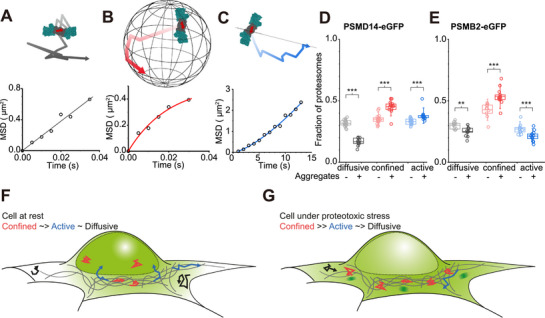
Mean‐squared displacement (MSD) analysis of SPT data. Individual tracks recorded from PSMB2‐eGFP or PSMD14‐eGFP molecules were classified as A) free (stochastic) diffusion, B) confined diffusion, or C) active transport. D) The fraction of molecules in each cell that was best described as free diffusion, confined diffusion, or active transport are plotted from resting cells expressing PSMD14‐eGFP (*n* = 18 cells and 33 380 tracks), and cells incubated with aS aggregates (*n* = 14 cells and 25 679 tracks). E) Similarly, resting cells expressing PSMB2‐eGFP (*n* = 11 cells and 35 312 tracks) and after incubation with aS aggregates (*n* = 10 cells and 14 158 tracks) were also characterized by MSD analysis. F) Schematic model comparing the global proteasome reorganization of cells at rest, and G) under proteotoxic stress. Here, proteasomes in resting cells are predominantly localized to the nucleus (green shading). Cells under proteotoxic stress re‐balance their proteasome distribution, and can form TAADs (dark green shading). Individual proteasomes are increasingly confined (red tracks) in cells under proteotoxic stress compared to resting cells, while the fraction of proteasomes that can freely diffuse (black tracks) are significantly reduced. Active transport (blue tracks) of RP and CP show slight increases and decreases, respectively, during proteotoxic stress.

We anticipated that aggregate clearance would be a slow process^[^ ^1^ ^]^ and reasoned that TAADs would enable continuous disaggregation and degradation,^[^ ^34^, ^35^ ^]^ which is supported by our recent work.^[^ ^20^ ^]^ To demonstrate this, we computed the SPT of individual tracks to calculate the mean square displacement (MSD) over time. We fitted individual MSD plots for each particle to a model that enabled their characterization either as a freely (stochastic) diffusing particle, a diffusive particle confined in a “molecular cage,” or a particle undergoing directed motion/active transport (see Experimental Section). Our data showed that the cellular fractions of proteasomes that matched those models were in 3:4:3 ratio, respectively (**Figure** **5**A–C). Comparing between resting and aggregate‐stressed conditions in PSMD14‐eGFP cells, we observed an increase in the fraction of proteasomes confined within molecular cage, while the fraction of freely diffusing particles reduced (Figure 5D). A less pronounced increase in proteasomes traveling by active transport was also observed (Figure 5D), consistent with cytoskeleton‐dependent motion of proteasomes in response to stress stimuli suggested in Figures 2 and 4. Since the eGFP tag reflected RP motions that may or may not be capped by CPs, our observation did not distinguish between the transport of free RPs or assembled holoenzymes.

We therefore repeated these experiments in PSMB2‐eGFP cells to follow CP motions, and again observed a strong increase in caged proteasome movement upon aggregate incubation (Figure 5E). This occurred concomitantly with a decrease in the fraction of freely diffusive proteasomes. Unlike observations made with RPs, the fraction of CPs under active transport was slightly reduced following aggregate incubation (Figure 5E). Since aggregate incubation did not alter the ratio between the different forms of proteasomal particles (see Figure 1G,H), our results in Figure 5A–E suggest that more of free RPs and less of free CPs are found under active transport during proteotoxic stress.

To determine the boundaries of the molecular cage and therefore confined diffusion, we modeled the radius of confinement from fitted trajectories, which found that proteasomes were caged within a volume with an approximate radius of 0.39 ± 0.04 µm (see Experimental Section). Proteasomes within that volume would be slow‐moving. Interestingly, incubation with aS aggregates decreased the size of confinement in stressed cells when compared to confined proteasomes in resting cells, but not with tau aggregates (Figure S11, Supporting Information). The difference in radius of confined diffusion may be triggered by differences in aggregate size and morphology assembled from each protein. Alternatively, proteasomes may recruit different additional proteins, some of which we have shown to be present in TAADs,^[^ ^20^ ^]^ to support the clearance of aS versus tau aggregates.

Taken together, our data suggest a model for proteasome motion in cells (Figure 5F), which at resting state maintains a balance between active transport, freely diffusive, and confined movement. While active transported and freely diffusive proteasomes are equally likely to be detected, confined movement of proteasomes is slightly more common than the other two. Upon aggregate internalization, a careful reorganization is orchestrated to predominantly increase the proportion of proteasomes in confined movement (Figure 5G).

We validated this model in differentiated SH‐SY5Y neuroblastoma cells CRISPR‐engineered to express PSMB2‐eGFP from the genomic loci and incubated these with aS aggregates. TAADs were confirmed to be cytoskeleton‐dependent, as treatment with Colcemid prevented their formation in SH‐SY5Y cells (Figure S12A, Supporting Information). SPT experiments further demonstrated that incubation with aS aggregates led to a pronounced reduction in proteasomal translocation, which was found more frequently confined during proteotoxic stress and consistent with observations in Figure 5A–E (Figure S12B–D, Supporting Information). We therefore propose, based on these results, that with more proteasomes in confined movement, this increases the chance of engaging and trapping of aggregates, most evidently by the formation of TAADs.

## 3 Discussion

While both diffusional movement and active transport of proteasomes have been reported previously,^[^ ^29^, ^31^, ^36^ ^]^ we demonstrated in this study how proteasomes respond to stress by altering their mode of motion to effectively increase proteasome concentration and forming TAADs at sites of degradation. This would enable increased degradation activity, e.g. against protein aggregates. Our findings suggest that the distribution and motion of CPs and RPs were close to identical and could not be quantitatively distinguished by localization or stress‐induced reorganization. It is therefore very likely that proteasome holoenzymes remain assembled at a stable level in equilibrium with free CP and RPs.

Slow‐moving proteasomes were previously attributed to possible interactions with other large macromolecules.^[^ ^31^ ^]^ We further expand this view and suggest that under changing cellular conditions, proteasomes may also associate reversibly with cellular organelles^[^ ^13^, ^36^, ^37^ ^]^ or accumulate together to form cellular bodies, which could account for the slow‐moving population. Our results further show that the balance between the three modes of proteasome motion: confined diffusion, free diffusion, and active transport is carefully modulated and tuned to specific cellular requirements. This enables proteasomes to reorganize upon proteotoxic stress, increasing the proportion of proteasomes confined in TAADs and at other centers of degradation.^[^ ^18^, ^38^, ^39^, ^40^ ^]^ By allowing reorganization of proteasomes, it follows that the availability of degradation activity is also redistributed during aggregate clearance, thus likely deprioritizing normal cellular proteostasis activities. However, delayed return of confined proteasomes to other subcellular regions may possibly contribute to the decline in protein degradation. This may become further aggravated should aggregates become degradation‐resistant and prolong proteasome confinement, thus impeding regular cellular proteostasis events. While we did not observe cells undergoing apoptosis, it will be interesting to explore the crosstalk between processes involved in the proteotoxic stress response and apoptotic pathways.

Similarly, the observed fraction of freely diffusing proteasomes under the resting state may also be by design to minimize time and energy redistributing proteasomes to nascent degradation centers, such as TAADs. For instance, stalling proteasomes from a reservoir of freely diffusing proteins may enable efficient targeting of aggregates than active transport of proteasomes located at distal sites. The observed changes in the proportion under active transport are relatively small compared to freely diffusing and confined proteasomes. As active transport requires ATP, this may suggest that cells will only expend energy on proteasome movement when it is absolutely required. Transporting proteasomes against concentration gradients would therefore only occur to fulfil acute proteostasis needs. Since the turnover of many proteins are controlled by degradation, it is reasonable to suggest that small changes in local proteasome levels may be sufficient to activate physiological processes sensitive to degradation. It remains to be discovered in future studies how distinct processes associated with, or regulated by proteasomal degradation may be affected by altering their distribution.

## 4 Experimental Section

### Transgenic Cell Lines

Genomic sequences of mammalian *PSMD14* and *PSMB2* were modified at the 3′‐end of the last exon with the CRISPR‐Cas9 genetic engineering system to replace the stop codon with DNA sequences coding for eGFP or mEos3.2. The final construct codes for a (GGS)×3 linker between the proteasomal subunit and the fluorescent protein (FP), and a myc‐ (for *PMSD14* editing) or Flag‐tag (for *PSMB2* editing) immediately C‐terminal to the FP. To edit the genome in HEK293A (HEK) and SH‐SY5Y cells, co‐transfection was performed with a donor plasmid, a gRNA plasmid (targeting the 3′‐end of either *PSMD14* or *PSMB2* DNA sequence), and an SpCas9 expression plasmid (Addgene #62 988). Four donor plasmids were created to generate the desired modifications, each encoding (GGS)×3‐eGFP‐myc or (GGS)×3‐mEos‐myc sequence flanked by the homology arms of *PSMD14*; alternatively, (GGS)×3‐eGFP‐flag or (GGS)×3‐mEos‐flag flanked by the homology arms of *PSMB2*.

Clones expressing eGFP or mEos were individually sorted by fluorescence intensity and grown to confluency. Correct insertions of the FP sequence into the genome were identified by PCR using primers based on the flanking sequences and confirmed by sequencing. Cell lines deemed suitable for TIRF imaging were selected for subsequent experiments. All cell lines used in this study are clonal and heterozygous at the modified locus, expressing both unmodified and FP‐modified proteasomal subunits. The FP was detected at a suitable level for single‐molecule imaging in cells.

### Western Blot Analysis of Cell Lysates

PSMD14‐eGFP, PSMD14‐mEos, PSMB2‐eGFP, and PSMB2‐mEos knock‐in cells were washed thrice in PBS, and collected by scraping in lysis buffer (50 mm Tris, pH 7.4 at 4 °C, 5 mm MgCl_2_, 2 mm ATP). Cells were lysed by homogenization using a motorized pestle mixer (431–0095, VWR) for 30 s and centrifuged on a benchtop centrifuge at 21 000 × g for 20 min at 4 °C. The supernatant was transferred to new tubes, and the concentration was measured by Bradford assay. Cell lysates (20 µg) were resolved on gels and transferred to 0.45 µm PVDF membranes (Bio‐Rad) using Trans–Blot semi‐dry Transfer System at 25 V, 2.5 A for 15 min. Membranes were then blocked in 5% BSA in Tris‐Buffered Saline with 0.05% Tween 20 (TBS‐T) for 1 hr at 4 °C, and incubated with primary antibody for PSMD14 (MA5‐35818, Thermo Fisher) or PSMB2 (sc‐58410, Santa Cruz) overnight at 1:1 000 dilution. Following three washes for 5 min in TBS‐T (20 mm Tris pH 7.6 at room temperature (RT, 22 °C), 150 mm NaCl, 0.1% Tween 20), membranes were then incubated with secondary antibodies Goat anti‐rabbit Alexa Fluor 488 (A‐11 008, Thermo Fisher) or Goat anti‐mouse Alexa Fluor 647 (A‐21 235, Thermo Fisher) at 1:10 000 dilution for 1 hr at RT. Finally, membranes were washed thrice for 10 min in TBS‐T before fluorescence of secondary antibodies was detected on an iBright imaging system (Invitrogen).

### SH‐SY5Y Maintenance and Differentiation

SH‐SY5Y human neuroblastoma cells were maintained in Dulbecco's modified Eagle's medium (DMEM), supplemented with 10% (v/v) fetal bovine serum (FBS), at 37 °C in 5% CO_2_, and passaged using 0.05% Trypsin‐EDTA. To differentiate SH‐SY5Y cells, an established protocol was used.^[^ ^41^ ^]^ Briefly, cells were plated at a density of 10 k cm^−2^ on coverslips. The following day, media was changed to DM1 (DMEM (no sodium pyruvate) supplemented with 5% FBS and 10 µm retinoic acid) every day for three days. Cell media was changed to DM2 (Neurobasal A, supplemented with N2 (1X), L‐glutamine (1 mm), and BDNF (50 13 ng µL^−1^)) every day for a further three days.

### Alpha‐Synuclein and Tau Aggregation

Recombinant aS and full‐length tau P301S mutant (isoform 0N4R) were purified following protocols as described before.^[^ ^34^, ^35^, ^42^ ^]^ Aggregation of aS was completed at 70 µm in reaction buffer containing 50 mm Tris, 150 mm NaCl, pH 7.4, 0.1% NaN_3,_ incubated at 37 °C, shaking at 200 rpm for 72 hrs. Tau aggregates were formed using 2 µm tau monomers in PBS, incubated with an equimolar final concentration of low‐molecular‐weight heparin (average 5 kDa; Thermo Fisher Scientific) at 37 °C for 24 hrs without shaking. Aggregated samples were sonicated for 15 min prior to incubation with cells.

### Cell Imaging Preparations

Cells were plated on glass coverslips (0.17 mm, Thorlabs) and grown at 37 °C in DMEM (Sigma) containing 10% FBS and 1% penicillin‐streptomycin one day before imaging. All glass coverslips were cleaned with Argon plasma for 1 hr before incubation with cells. For SMLM imaging of mEos localizations, fixed cells were incubated with PBS buffer containing 4% paraformaldehyde (PFA) and 0.2% glutaraldehyde for 10 min on the bench. Prior to imaging, cells were washed thrice and imaged in warm OptiMEM (Sigma) pre‐filtered through 0.02 µm pores. A metal chamber was used to secure the coverslip above the objective. All imaging was performed in temperature‐controlled chambers. For microtubule or actin depolymerization assays, cells were incubated with 5 µm Colcemid (Sigma) or Latrunculin A (Anachem), prepared in DMSO or ethanol, respectively. After 10 min at 37 °C, cells were taken out from the incubator and washed three times using OptiMEM before imaging as described above.

### Light‐Sheet Imaging

Cells were imaged using single objective cantilever SPIM (socSPIM), as detailed previously.^[^ ^43^ ^]^ Briefly, a commercial AFM cantilever (ContAl‐G, BudgetSensors) was positioned above the objective of an inverted microscope (Eclipse TiU, Nikon). The cantilever was coated with reflective aluminium and was fitted onto a machined brass rod using cyanoacrylate adhesive. A cylindrical lens placed before the cantilever generated a light sheet with an axial thickness of ≈1.9 µm full width at half maximum. The sample stage was moved by a xyz piezo (P‐611.3 Nanocube, Physik Instrumente), allowing the light‐sheet to scan through the sample at 30 ms exposure time with scanning steps of 200 nm. Scanning data was assembled into hyperstacks and rendered using the 3D projection plugin and 3D viewer plugin in ImageJ/FIJI.

### Immunofluorescence Labeling to Assess Relative Fractions of Singly‐ or Doubly Capped Proteasomes

Cells were fixed with 4% PFA in PBS (20 min at RT). Cells were washed thrice in PBS, and permeabilization was performed with Triton (0.1%, in PBS, 15 min at RT). Blocking was performed for 1 hr at RT with 10% (v/v) goat serum (GS) in PBS. Primary antibody incubation was performed in 5% GS overnight at 4 °C. After three washes with PBS, secondary antibody incubation (5% GS in PBS) was performed for 1 hr at RT, protected from light. Cells were washed three times with PBS prior to imaging. Primary antibodies against RP (rabbit anti‐PSMD14, 1:400), CP (mouse MCP21, 1:400) and secondary anti‐rabbit‐Alexa568 (1:1 000) and anti‐mouse‐Alexa488 (1:5 000) were used. Monomers of aS were also labeled with Alexa647, and were aggregated with unlabeled monomers at a 1:9 ratio labeled: unlabeled under shaking conditions and sonicated, as previously described, to produce labeled alpha‐synuclein aggregates capable of passing through cell membranes.^[^ ^1^ ^]^ Cells were incubated with 1 µm labeled aggregates for 24 hrs before cells were fixed and/or imaged.

### Labeling of Endoplasmic Reticulum by Transient Expression of eGFP‐Sec61

Cells endogenously expressing PSMD14‐mEos were plated on plasma‐cleaned coverslips. Subsequently, cells were transfected with the eGFP‐Sec61 plasmid using JetOptimus (Polyplus) following the manufacturer's protocol. Media was replaced 24 hrs post‐transfection, and cells were treated with 1 µm aggregates for 24 hrs as described above. All coverslips were imaged 48–72 hrs post‐transfection. Photoconversion of mEos particles was achieved through illumination of the sample using a short pulse of the 405 nm laser immediately prior to imaging. mEos was imaged with 561 nm excitation, eGFP with 488 nm excitation, and Alexa647 with 638 nm excitation.

Annexin V expression was determined by immunostaining of HEK293A cells prepared as above. Cells were incubated with monoclonal anti‐Annexin V (6A12, Abcam) in 1:200 dilution for 1 hr at RT and washed thrice with PBS. This was followed by 30 min incubation with secondary anti‐mouse‐Alexa568 with 1:1 000 dilution at RT. Cells were given a final three washes with PBS before imaging.

### SMLM Imaging of Immunofluorescence‐Labeled Cells

Cells were imaged using an ECLIPSE Ti2‐E inverted microscope (Nikon), coupled to a sCMOS camera (Prime95B, Photometrics) and C‐FLEX laser combiner (HÜBNER Photonics), as previously described.^[^ ^1^ ^]^ Briefly, imaging of fixed cells was completed by incubating cells with imaging buffer (PBS with 1 mg mL^−1^ glucose oxidase, 0.02 mg mL^−1^ catalase, 10% (w/w) glucose, and 100 mm methylamine). Each field of view was recorded as a set of 2000‐frame movies sequentially for each fluorophore. Multicolor images of each field‐of‐view were reconstructed to a single frame using Matlab scripts.^[^ ^44^ ^]^ Bursts of fluorescence from individual molecules were identified after applying a box filter and thresholds for each pixel, which were used based on that specific pixel's variance when measuring background noise. Single‐molecule localizations from each frame were calculated by fitting the point‐spread function (PSF) to a 2D Gaussian profile, and the background and signal intensities of these PSFs are shown in Figure S13 (Supporting Information). Chromatic aberrations were corrected using a second‐order polynomial function, as determined by imaging Tetraspeck beads (0.1 µm, fluorescent blue/green/orange/dark red; Life Technologies) across the emission wavelengths used in the experiment. Localizations from all frames and colors were aligned and plotted to produce images with planar resolutions of 21 ± 1 nm.^[^ ^1^ ^]^

### HILO 3D Imaging of Cells

Cells were imaged using the ECLIPSE Ti2‐E inverted microscope (Nikon) described above. The imaging buffer for observing live cells was Fluorobrite supplemented with 10% FBS. The E‐TIRF arm was used to position the laser beam to illuminate the sample at an angle suitable for HILO imaging. The objective was moved axially in steps of 100 nm to image the entire cell volume, and 3D representations of each cell were rendered using the 3D viewer Image/FIJI plugin.

### Image Analysis to Calculate Fractions of Singly‐ and Doubly‐ Capped Proteasomes

Regions of interest were identified within multicolor reconstructed SMLM images of cells labeled with fluorescent antibodies against CPs and RPs, respectively. Each region of interest was analyzed using the ImageJ/FIJI plugin ComDet (v.0.5.5). Holoenzymes were identified as CP and RP clusters within 100 nm of each other. To determine the number of RPs in each holoenzyme, the RP fluorescence intensities for each cluster in the cell were used to calculate the average intensity of a single RP. The number of singly‐ and doubly‐capped holoenzymes was calculated and is reported as fractions of the total number of CP or RP clusters, respectively.

### Nanopipette Delivery and Imaging of aS Aggregates

Nanopipettes were fabricated from quartz capillaries to achieve ≈300–400 nm inner and ≈600–800 nm outer diameter with 40–80 MΩ electronic resistance, detailed elsewhere.^[^ ^27^ ^]^ Briefly, the nanopipette was backfilled with 8 µL of 1.4 µm aS monomers or aggrega in PBS, with an Ag/AgCl electrode inserted inside. The pipette was immersed in a bath of PBS buffer, which contained cell samples, while another Ag/AgCl electrode served as the reference electrode was put into the buffer. A +100 mV voltage bias was applied between the electrodes inside the nanopipette and the bath, generating a +2 nA ionic current. Prior to injection, the nanopipette was first moved down to a position ≈15 µm above the cell surface using the white light microscope, and the piezo moved the nanopipette down to the target area at a speed of 25 µm s^−1^, while the ion current was recorded in a field programmable gate array (FPGA) at the same time. Once the nanopipette had approached the cell surface, the piezo moved the pipette down by an extra 5 µm in one step to penetrate the cell membrane. Once the nanopipette had penetrated the cell membrane, a voltage pulse (−500 mV, 10 s) was applied to force the molecules out of the nanopipette and into the cytoplasm. After the injection process was finished, the nanopipette was retracted back by 5 µm in one step preventing the penetrated cell sticking on the nanopipette and moving up with it.

### Microtubule Imaging

PSMD14‐eGFP cells were incubated at 37 °C for 24 hrs with 1 µm aS aggregates. Untreated cells or cells treated with 0.25 µm Colcemid or LatA were incubated with 1 µm SiR‐tubulin (Spirochrome) for 30 min prior to imaging. The cell culture media was then replaced with 0.1 µm SiR‐tubulin in warm, pre‐filtered OptiMEM, maintaining the Colcemid and LatA concentrations. The cells were imaged in HILO mode as described above.

### Imaging in TIRF and HILO for Single‐Particle Tracking

Imaging of live cells was performed using a bespoke TIRF microscope. Diode lasers operating at 405 nm (Cobolt MLD), 488 nm (Toptica) or 561 nm (Cobolt Jive 500) were directed into an oil‐immersion TIRF objective (60× Apo N TIRF, NA 1.49, Olympus) mounted on an Eclipse Ti2 microscope (Nikon Corporation). The change from HILO to TIRF mode was achieved by altering the beam offset perpendicular to the optical axis, resulting in TIRF/HILO imaging. Emitted fluorescence was collected by the same objective and separated from the returning TIR beam by a dichroic filter (Di01‐R405/488/561/635‐25×36; Semrock). Emission filters were applied for eGFP (FF01‐496/LP‐25, FF01‐520/44‐25) or mEos (BLP02‐561R‐25, FF01‐607/36‐25) imaging. Images were recorded on an EMCCD camera (Evolve 512Delta, Photometrics), by alternating between laser excitation sources (561 nm laser at 0.8 kWcm^−2^, 488 nm at 1.5 kWcm^−2^ and 405 nm at 0.7 kWcm^−2^, determined by measuring the power exiting the objective and the footprint of the beam in the imaging plane) using mechanical shutters (Prior Scientific) and an exposure of 55 ms for localization microscopy. For single‐molecule tracking, the measured exposure time was 5 ms. A short pulse of 405 nm was used to photoconvert mEos, which was subsequently excited using the 561 nm laser. The pixel size (107 nm) was measured prior to recordings and the precision in the XY plane was calculated as 25 ± 10 nm, as described by Weimann et al.^[^ ^32^ ^]^

### Single‐Molecule Tracking Analysis

Single‐molecule images were analyzed using a custom Matlab script.^[^ ^32^ ^]^ A bandpass filter (1 to 5 pixels) was first applied, followed by the identification of local maxima in raw images. Maxima were identified based on a signal‐to‐noise threshold, which was set to 4× the standard deviation above the background intensity, and spots with a radius less than 8 pixels were then linked together to form tracks.^[^ ^45^ ^]^ The tracking algorithm allowed molecules to move a maximum distance of 6 pixels (8 pixels for eGFP‐USP21) per frame (5 ms). Only tracks longer than 6 frames were used for analysis.

### Diffusion Analysis by Jump Distance

Jump distance (JD) analysis has been described before^[^ ^32^, ^46^ ^]^ and was applied to tracks detected from each imaged cell to investigate heterogeneities in the diffusion coefficient distribution. Briefly, the probability distribution *P*(*r* ^2^,Δ*t*) describing the probability of a particle travelling a distance between *r* and *dr*, t in one time step, *Δt*, was fitted using Equation (1):

(1) $$P\left(r^{2},\Delta t\right)=\underset{j=1}{\sum^{m}}\;\frac{f_{i}}{4D_{j}\Delta t}e^{-\frac{r^{2}}{4D_{j}\Delta t}}$$

where *f* is the fraction corresponding to population *j*, and *m* is the number of populations. *JD* analysis was applied to tracks from each cell individually. *m* was selected by evaluating the coefficient of determination, *R^2^*, of the fit to a single population. *m═1 for R^2^>0.9* and *m═2 for R^2^<═0.9* (detailed in ref. [32]).

### Diffusion Analysis by Mean Square Displacement

The MSD curve for each single particle track was calculated using Kehl,^[^ ^47^ ^]^ a Matlab function written to calculate MSD plots avoiding using loops. MSD curves were calculated from SPT tracks using the sub‐pixel localization of particles at time *t* and *t* + τ. All displacements with the same value of τ are used to calculate a mean square displacement, and a MSD plot is recorded as a function of time intervals, τ, as described by Equation (2):

(2) $$MSD\left(\tau \right)=\langle {\left(x\left(t\right)-x\left(t+\tau \right)\right)}^{2}\rangle$$

Each particle's behavior was classified as either freely diffusing, diffusing in a cage, or being actively transported according to methods based on Otero et al.^[^ ^36^ ^]^ Briefly, the MSD plot is fitted using Equation (3):

(3) $$MSD\left(\tau \right)=A\tau^{\alpha }+B$$

where α can vary between 0 and 2. Particles were described as freely diffusing if α < 0.5, confined in a cage if 0.9 < α < 1.1, or being actively transported if α > 1.25 (particles were discarded if α lies outside these ranges). The fraction of particles within each cell, *n*, behaving as freely diffusive, confined, or under active transport, was calculated using Equation (4):

(4) $$Fraction_{n}=\frac{Particles_{n}}{Total\;\;Particles}$$

The radius of confinement was calculated by fitting each MSD curve classified as a confined particle to Equation (5):

(5) $$MSD=r_{c}^{2}\left(1-e^{-\frac{x}{b}}\right)$$

where $r_{c}^{2}$ represents the plateau of the MSD curve, and the radius of confinement (*r_c_*) for each particle. The mean radius of confinement for all particles in each cell is plotted in Figure S11 (Supporting Information).

### Patch Clamp and Imaging

Recording pipettes (4–6 MΩ) were pulled from borosilicate glass, and were filled with an internal solution (135 mm potassium gluconate, 10 mm HEPES, 10 mm sodium phosphocreatine, 4 mm KCl, 4 mm MgATP, 0.3 mm Na2GTP, pH 7.2 [adjusted with KOH]) and osmolarity set to 291 mOsmol l^−m^. Signals were acquired using an Axon Multiclamp 200B amplifier (Molecular Devices). Recordings were low‐pass filtered at 2 kHz and acquired at 5 kHz using an NI X Series Multifunction DAQ (National Instruments) programmed by Axograph software. Membrane voltage was recorded in the current clamp mode. Series resistance was 10–25 Mso and experiments were terminated if this range was exceeded.

To investigate proteasomal responses to membrane potential, the membrane potential of cells was manipulated in voltage clamp mode with a 30 s step (unless otherwise specified) of −100 or +100 mV alternatively. Current traces were filtered at 2 kHz and digitally sampled at 20 kHz using AxoGraph software. The bath level of the recording chamber was kept to a minimum to reduce pipette capacitance. Leak current was subtracted off‐line using a template constructed from the currents induced by a −10 mV hyperpolarizing step. The resting V_m_ was found to be ≈−20 mV, consistent with published values for HEK cells (e.g., ^[^ ^48^, ^49^ ^]^). V_m_ was then clamped on cells expressing PSMB2‐eGFP or PSMD14 and oscillated between depolarization (+100 mV) and hyperpolarization (−100 mV). A lower laser power was used for TIRF illumination to reduce the rate of eGFP photo‐bleaching during patch clamp experiments.

White‐light transmission and fluorescence images were acquired with a modified fluorescent microscope based on an inverted microscope, equipped with an infinity‐corrected oil immersion objective (Olympus UPlanApo, 100×, NA 1.4), operating in TIRF imaging mode to reduce the excitation volume, and detected on a 512 × 512 pixel EMCCD (Evolve 512Delta, Photometrics) at a rate of 60 ms per frame for proteasome imaging. The filters used were a dichroic mirror (Di01‐R405/488/561/635‐25x36, Semrock) and a 525/50 nm band pass filter (FF03‐525/50‐25; Semrock). Laser excitation was provided by a 488 nm solid‐state laser (Spectra‐Physics) at a power density of ≈0.7 kW cm^−2^.

### Potential Regulated Imaging Analysis

Image Analysis was done using Fiji and custom‐written Matlab code. Image stacks were bleach‐corrected using the in‐built exponential *bleach correction* function of Fiji (Kota Miura et al., 2014). ImageJ Plugin CorrectBleach V2.0.2. Zenodo. 10.5281/zenodo.30769) applied to the cell of interest. Subsequently, the stacks were 10‐fold binned in time to generate frame rates of 600 ms. For the image analysis, time‐traces from the cell of interest were low‐pass filtered at a pass band frequency of 0.05 Hz, a steepness of 0.85, and stop band attenuation of 30 dB. The resulting traces were then normalized to their maximum intensities and analyzed using the Matlab function *findpeaks*, to detect maxima and minima with a prominence of 3% and a minimum peak distance of 50 frames. The rate of intensity increase per cell was measured as the intensity increase over the time between the first minimum to the closest maximum. If there was only one peak detected, the rate is calculated as the intensity increase over the time between the peak and the local maxima or minima. If there were no peaks detected, the rate is calculated as the rate of signal increase over the minimum to the maximum of the entire trace. Every cell was therefore assigned a rate of intensity increase in relation to the maximum intensity of that cell.

## Conflict of Interest

The authors declare no conflict of interest.

## Supporting information



Supporting Information

Supplementary Video 1

Supplementary Video 2

Supplementary Video 3

Supplementary Video 4

Supplementary Video 5

Supplementary Video 6

Supplementary Video 7

Supplementary Video 8

Supplementary Video 9

Supplementary Video 10

## Data Availability

The data that support the findings of this study are openly available in Redistribution of proteasomes through diffusion and cytoskeleton‐dependent mechanisms upon stress induced by protein aggregates at https://doi.org/10.1101/487702v2, reference number 487702.
