# Supplementary material for: Stress‐Induced Reorganization of Proteasomes Through Diffusion and Cytoskeleton‐Dependent Mechanisms
Source: Small. 2025 Nov 24;22(2):e06260. doi: 10.1002/smll.202506260 (PMC12781625; doi:10.1002/smll.202506260)
Supplement: Supplementary file 1 — Supporting Information [file SMLL-22-e06260-s010.pdf]

NANO · MICRO
small

Supporting Information

for *Small*, DOI 10.1002/smll.202506260

Stress-Induced Reorganization of Proteasomes Through Diffusion and Cytoskeleton-Dependent Mechanisms

*Michael J Morten, Yu Zhang, Bing Li, Jonathan X Meng, Kun Jiang, Liina Sirvio, Ji-Eun Lee, Anna H Lippert, Matilda Burridge, Katiuska Daniela Pulgar Prieto, Alexander R Carr, Aleks Ponjavic, Steven F Lee, Daniel Finley, David Klenerman and Yu Ye**

Supplementary information for:

Stress-induced reorganization of proteasomes through diffusion and cytoskeleton-dependent mechanisms

Michael J Morten[1], Yu Zhang[2], Bing Li[2], Jonathan X Meng[2], Kun Jiang[2], Liina Sirvio[1], Ji Eun Lee[2], Anna H Lippert[2], Matilda Burridge[1], Katiuska Daniela Pulgar Prieto[1], Alexander R Carr[2], Aleks Ponjavic[2], Steven F Lee[2], Daniel Finley[3], David Klenerman[2], Yu Ye[1,2]*

[1] Department of Brain Sciences, Imperial College London, 86 Wood Lane, London W12 0BZ

[2] Department of Chemistry, University of Cambridge, Lensfield Road, Cambridge CB2 1EW

[3] Department of Cell Biology, Harvard Medical School, Longwood Avenue, Boston 02115, Massachusetts

*Correspondence to yu.ye1@imperial.ac.uk

Contents

Supplementary figures: S1-13

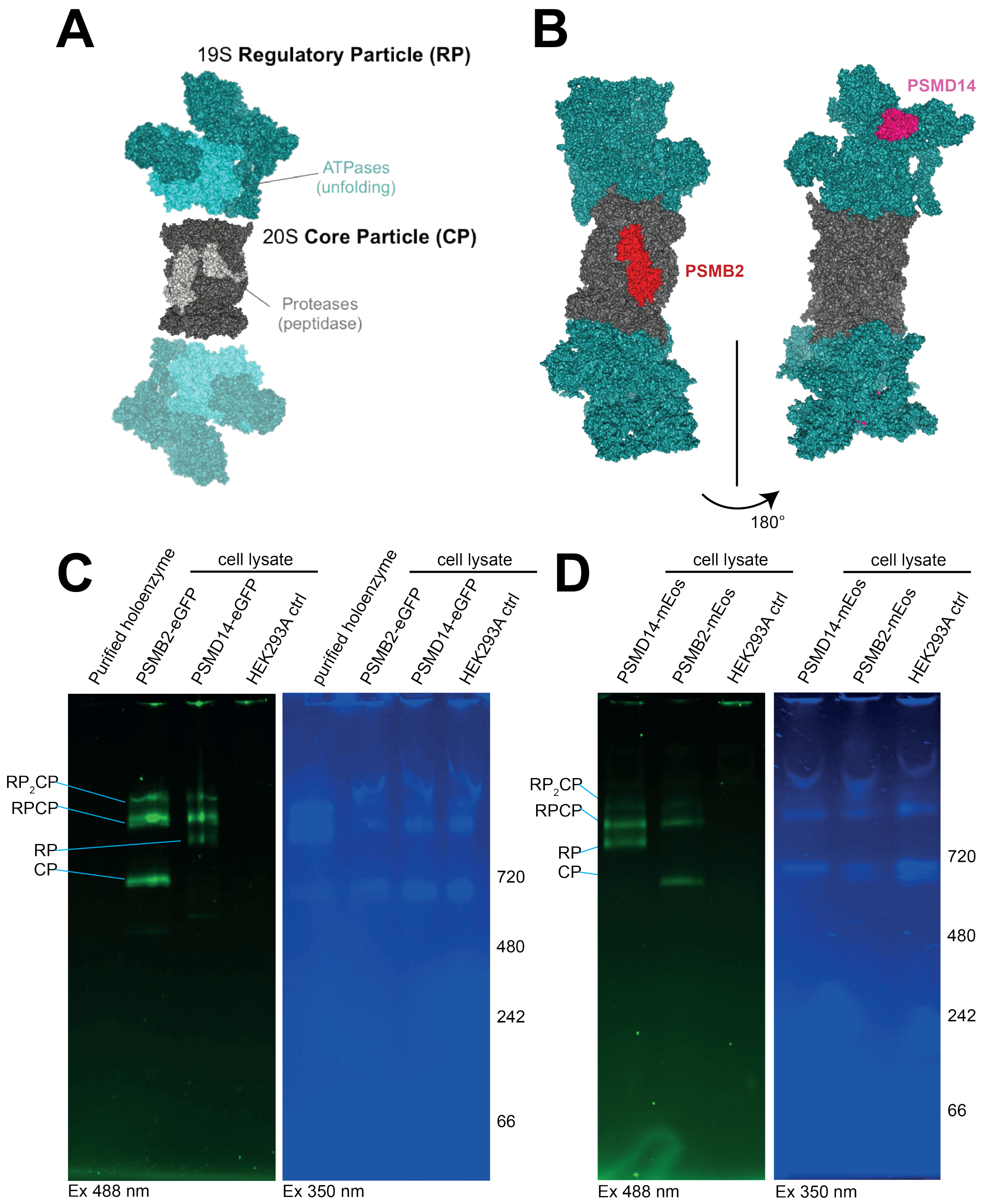

Figure S1. CRISPR-edited and FP-tagged PSMB2 or PSMD14 subunits expressed from their respective genomic loci are incorporated into proteasome particles. (**A**) Molecular structure of the mammalian 26S proteasome holoenzyme (pdb-id: 5GJQ) highlighting the protease subunits responsible for the peptidase activities of the 20S core particle (CP, in shades of gray) and the ATPases providing substrate unfolding and translocation activities of the 19S regulatory particles (RPs, in shades of cyan). (**B**) PSMB2 and PSMD14 subunits are highlighted (red) within the CP and RP, respectively, of the proteasome. Lysates from CRISPR-edited cell lines expressing (**C**) PSMB2-eGFP or PSMD14-eGFP, and (**D**) PSMB2-mEos or PSMD14-mEos are separated by native gradient-gel (4-16% acrylamide) electrophoresis and visualized by fluorescence excitation at 488 nm (*left*) for eGFP or mEos detection. Gels were incubated with

100 µM of suc-LLVY-AMC, a fluorogenic proteasome substrate, and visualized by excitation at 350 nm (*right*). Purified unlabeled 26S proteasome holoenzyme was loaded as a control and the bands corresponding to free CP, free RP and singly- and doubly-capped holoenzymes are indicated. Molecular weight markers are shown to demonstrate the absence of any free GFP-tagged subunits not incorporated into the proteasome.

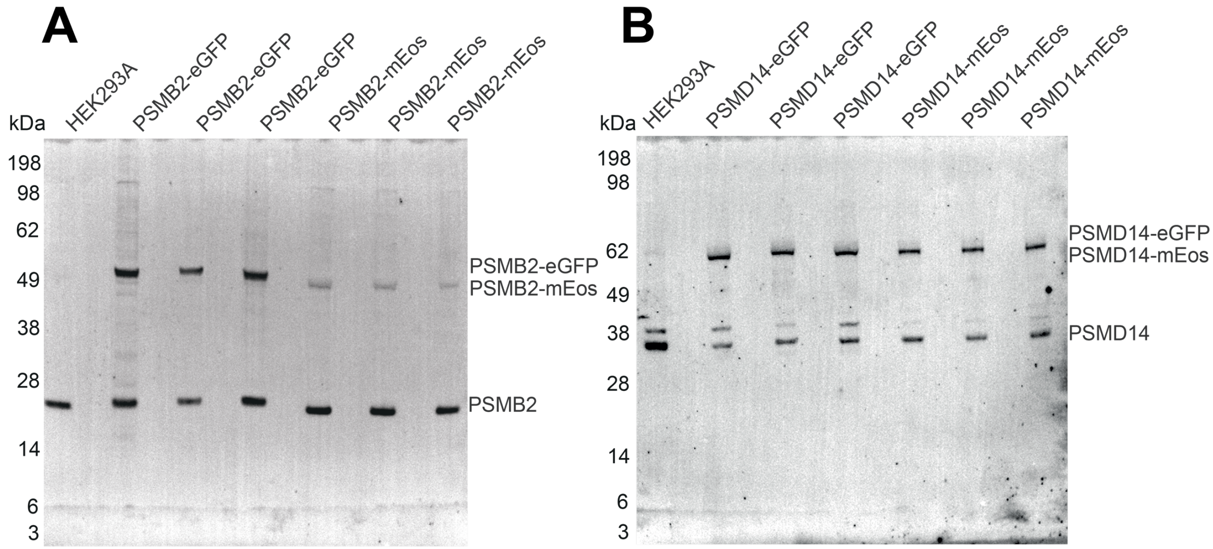

C

Cell line	Labeling percentage
PSMB2-eGFP	48%
PSMB2-mEos	12%
PSMD14-eGFP	59%
PSMD14-mEos	52%

Figure S2. Determining the percentage of endogenous proteasome subunits labeled by FPs. Western blots against (**A**) PSMB2 and (**B**) PSMD14 in three biological repeats each of cell lines expressing PSMB2-eGFP, PSMB2-mEos, PSMD14-eGFP or PSMD14-mEos. (**C**) Percentage of labeled proteasome subunits was determined from densitometry analysis of the FP-modified band as a percentage of total (FP modified + unmodified), averaging from three repeats rounded to the nearest integer.

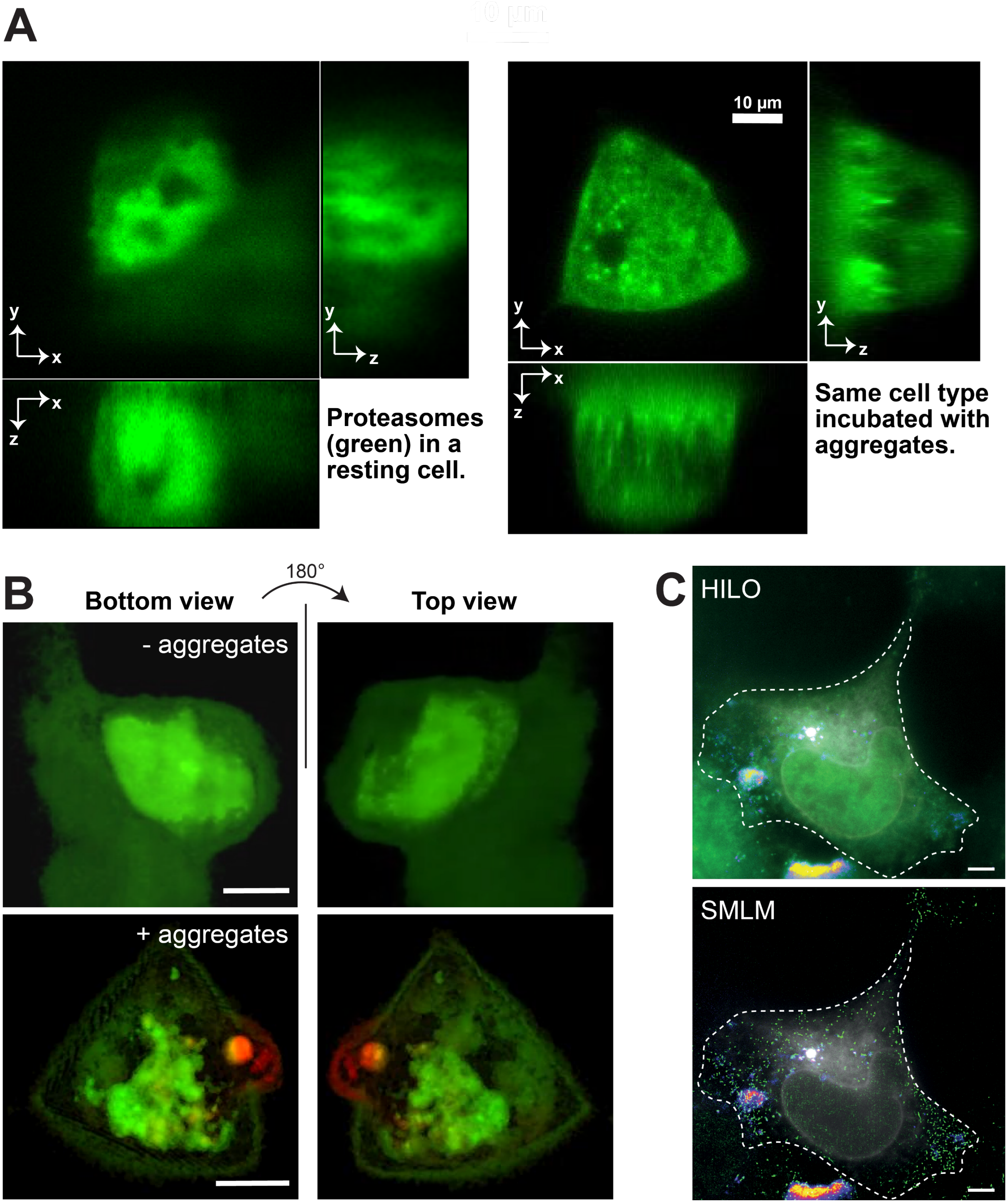

Figure S3. Light-sheet imaging validates that proteasome distribution changes upon aS aggregate invasion. (**A**) Light-sheet imaging of PSMD14-eGFP cells at rest (*left*) or treated with recombinant aS aggregates (*right*), performed using a bespoke setup previously described[39]. The change in distribution and the formation of proteasome foci are evident from the 3D image, shown as orthogonal views. Scale bar = 10 µm. (**B**) Two-color light-sheet imaging of resting cells (top) and cells incubated with Alexa647-labeled aS aggregates demonstrates co-localization of proteasome foci (green) and aggregates (red) inside cells. See **Materials and Methods** for details. Scale bar = 10 µm. (**C**) HILO and SMLM images of a typical cell expressing PSMD14-mEos (green) after 24 hrs incubation with 1 µM aS aggregates (fire-LUT). These cells also expressed eGFP-Sec61 (white) as an ER marker. Scale bar = 5 µm.

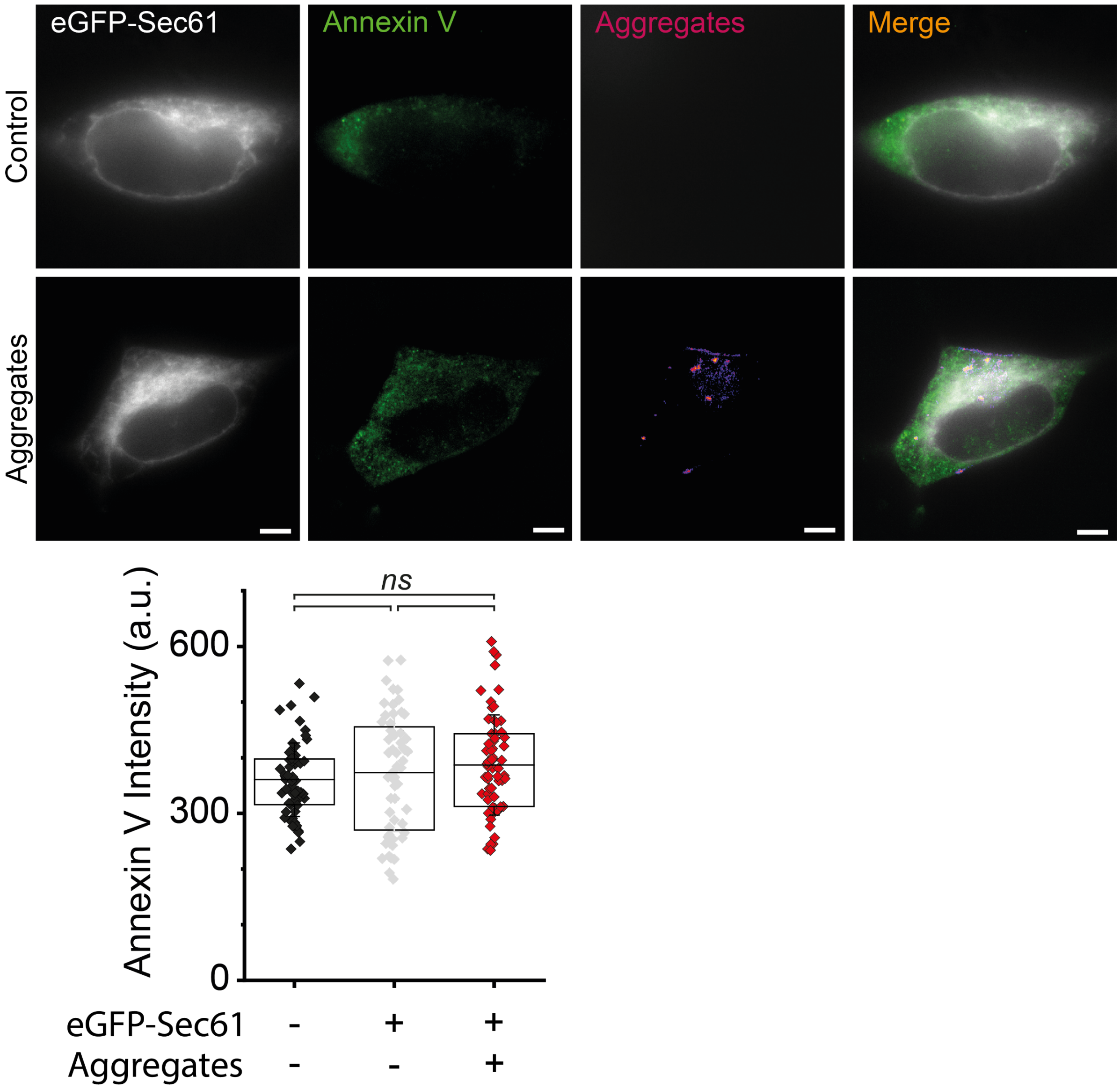

Figure S4. Immunofluorescence labeling of HEK293A cells expressing eGFP-Sec61 (white) with antibodies against Annexin V (green). Cells were incubated with aS-Alexa647 aggregates (fire-LUT) for 24 hrs and imaged under HILO conditions. The mean intensity of eGFP-Sec61 fluorescence for individual cells is plotted and shows no significant difference found between cells which were incubated with (n = 63 cells) and without (n = 56 cells) aS aggregates. These cells were also compared to cells not expressing eGFP-Sec61 without aS aggregates (n = 58 cells), and again no differences were observed. Box charts show the mean, and the 25th and 75th percentiles, with whiskers illustrating the standard deviation. Statistical significance was calculated using Student's *t-test*, where no significant difference (n.s.) $p>0.05$.

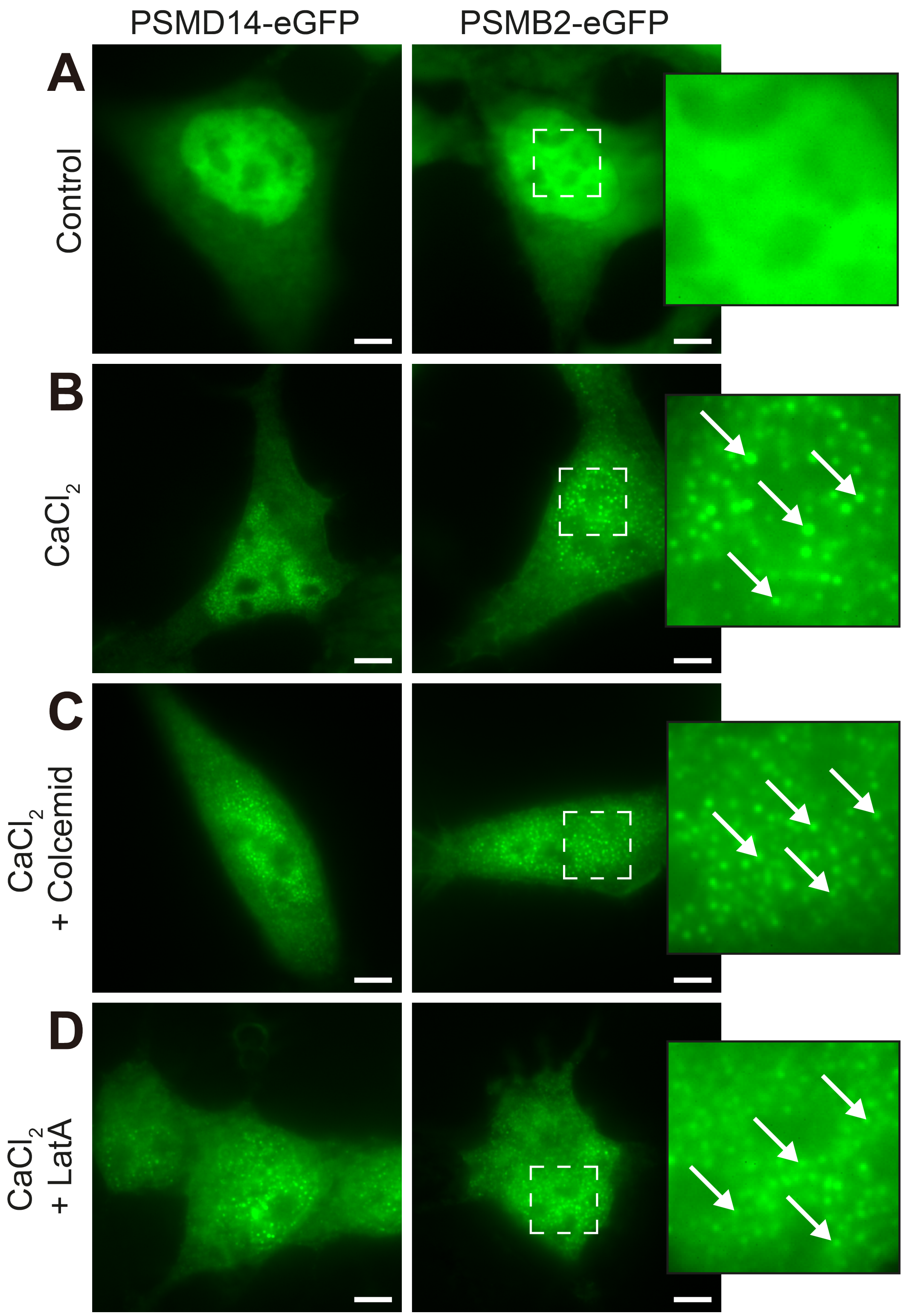

Figure S5. The formation of LLPS-foci induced by hypertonic stress is not cytoskeleton-dependent. (**A**) Untreated PSMD14-eGFP or PSMB2-eGFP cells were compared to (**B**) cells treated with 150 mM $CaCl_2$ for 10 minutes, with insets showing examples of LLPS-foci from PSMB2-eGFP cells, marked by white arrows. (**C-D**) The formation of the proteasome foci also occurred after pre-treatment of cells with 5 μM Colcemid or LatA prior to the addition of $CaCl_2$.

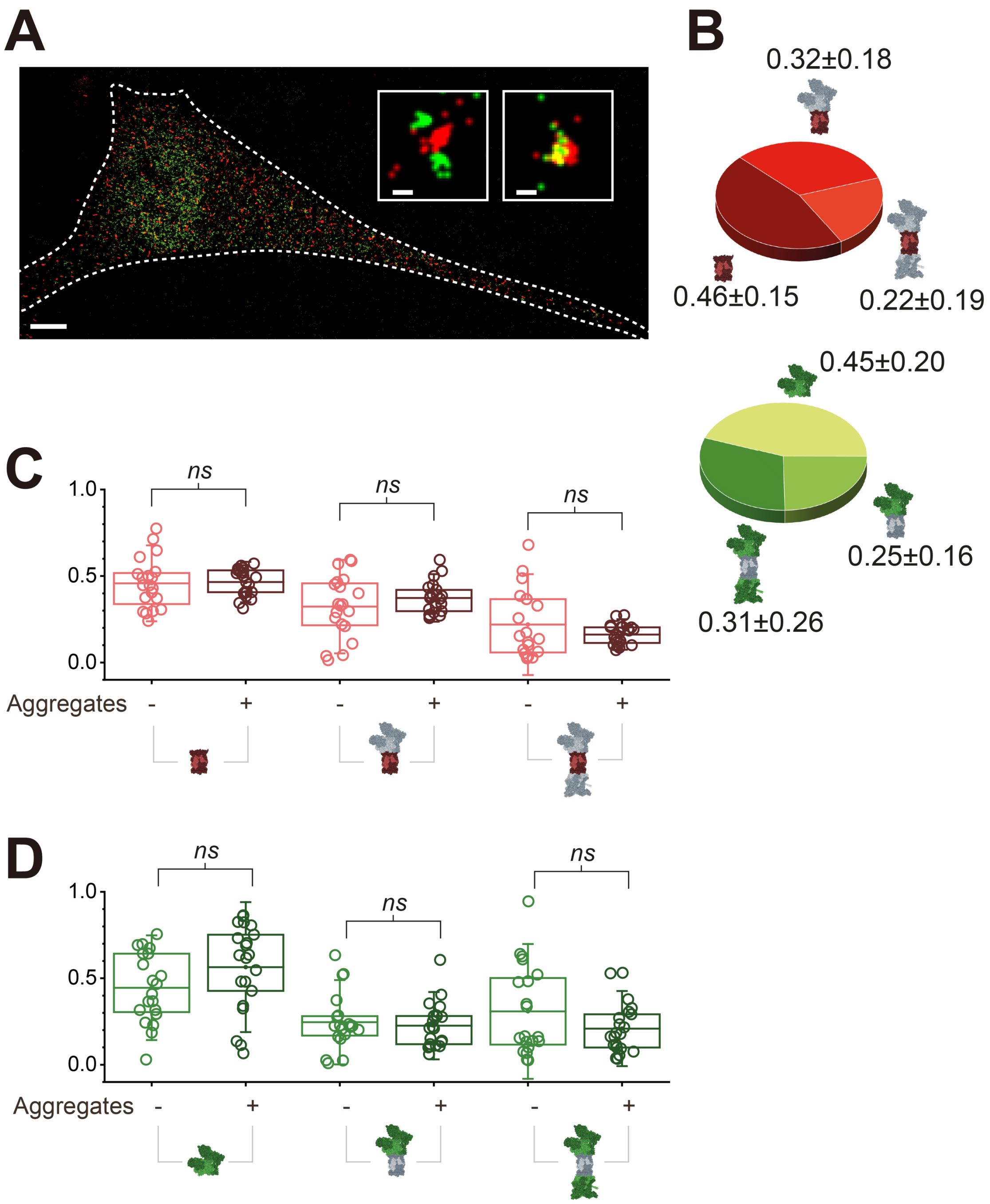

Figure S6. Immunofluorescence staining of differentiated SH-SY5Y cells shows free CP and RP in the cell and in complex. (**A)** Untreated SH-SY5Y cells were fixed and stained with antibodies to label CP (PSMB2, red) and RP (PSMD14, green). Insets show foci depicting singly- and doubly-capped proteasomes. Scale bars: 5 µm, and insets 50 nm. (**B**) Colocalization of CP (left) and RP (right) enabled calculation of uncapped versus singly- and doubly-capped proteasome ratios in resting cells. (**C**) CP and (**D**) RP box plots show that incubation with 1 µM aS aggregates do not induce any significant changes between the relative ratios of free particles or holoenzymes in the cytoplasm. (n = 20 resting cells and n = 21 cells incubated with aS aggregates).

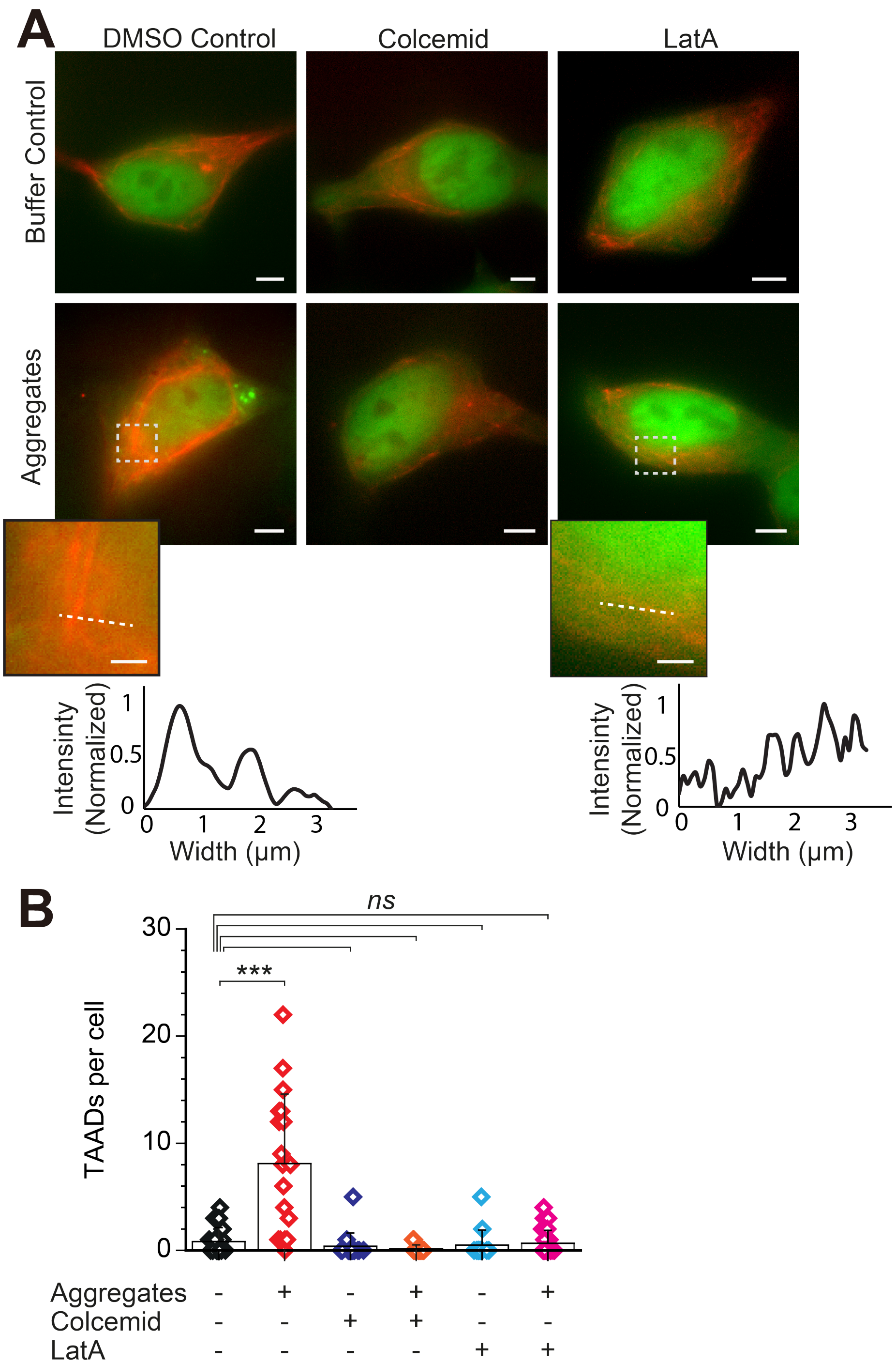

Figure S7. TAADs do not form in cells with disrupted microtubule networks. (**A**) HILO images of PSMD14-eGFP cells incubated with 1 µM aS for 24 hrs in the presence of 0.25 µM Colcemid or Latrunculin A (LatA). SiR-tubulin (red) was used to label microtubules within these cells. (**B**) Bar chart shows quantification of TAAD formation from the HILO images. TAADs form in control cells only in the presence of aS aggregates, whereas cells incubated with Colcemid or LatA did not form TAADs and showed less distinct networks of microtubules, as shown by the line profiles illustrating the fluorescence intensity of the SiR-tubulin marker. Scale bar: 5 µm and inset 1 µm. ($n_{control} = 19$, $n_{aggregates} = 18$, $n_{Colcemid} = 16$, $n_{Colcemid+Aggregates} = 14$, $n_{LatA} = 20$, $n_{LatA+Aggregates} = 24$.) Statistical significance was calculated using Student's *t-test*, where no significant difference (n.s.) $p>0.05$, * $p\leq0.05$, ** $p\leq0.01$ and *** $p\leq0.001$.

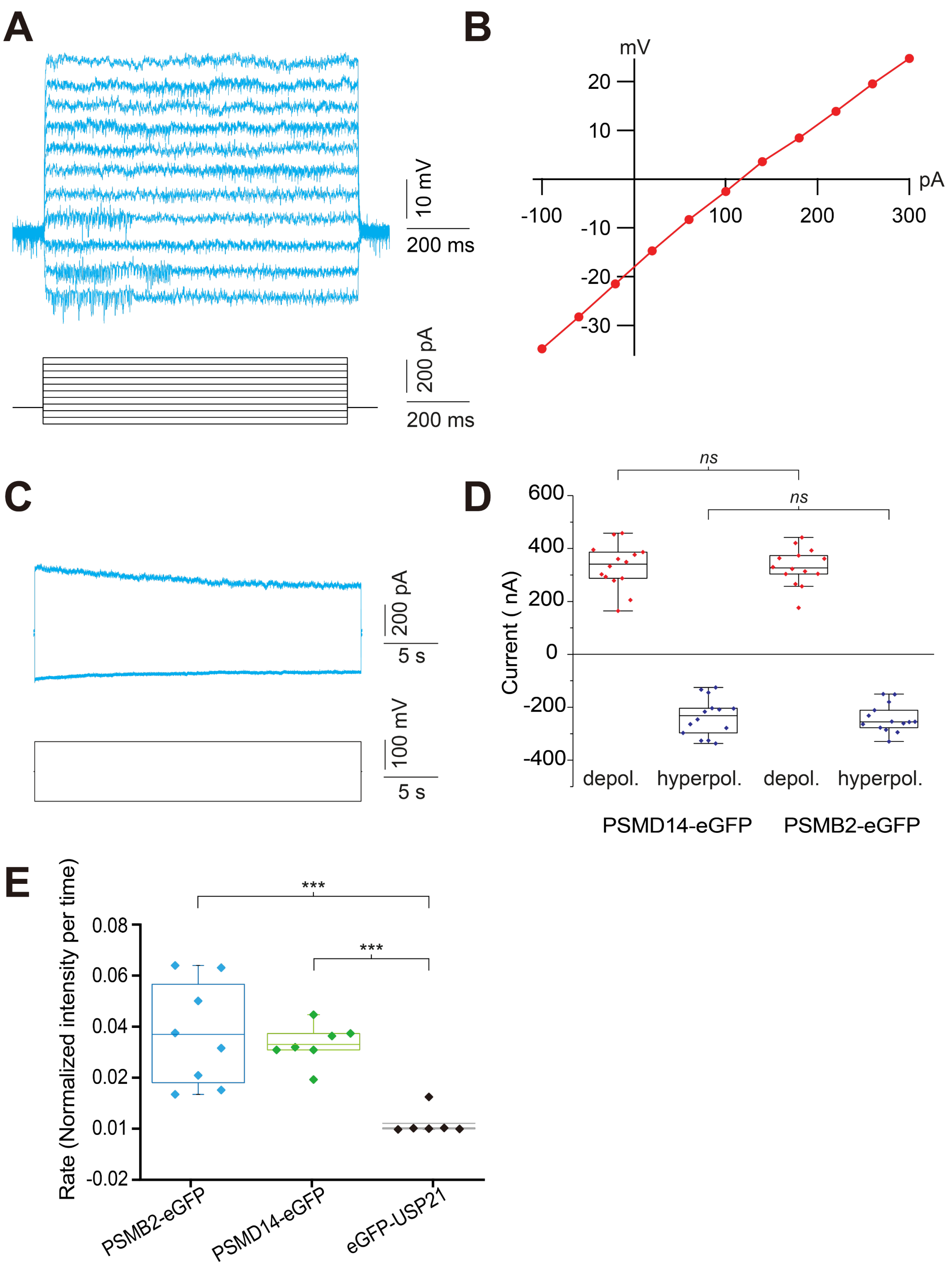

Figure S8. Current (I)-potential (V) relationship from patch-clamped HEK293A cells imaged by TIRF microscopy. (**A**) A typical set of voltage responses to current injections in a PSMB2-eGFP cell and (**B**) corresponding IV curve of a recorded cell. (**C**) Voltage control (+100 mV or -100 mV) of recorded PSMB2-eGFP cells with 30 s step plotted here against the current response to the voltage control. (**D**) Averaged current response range of HEK cells at holding potential +100mV or -100 mV (n = 14 cells). (**E**) Rate of change of normalized fluorescence intensity of cells expressing eGFP-USP21 with respect to cycling between depolarizing and hyperpolarizing membrane potentials, compared to PSMB2-eGFP and PSMD14-eGFP cells.

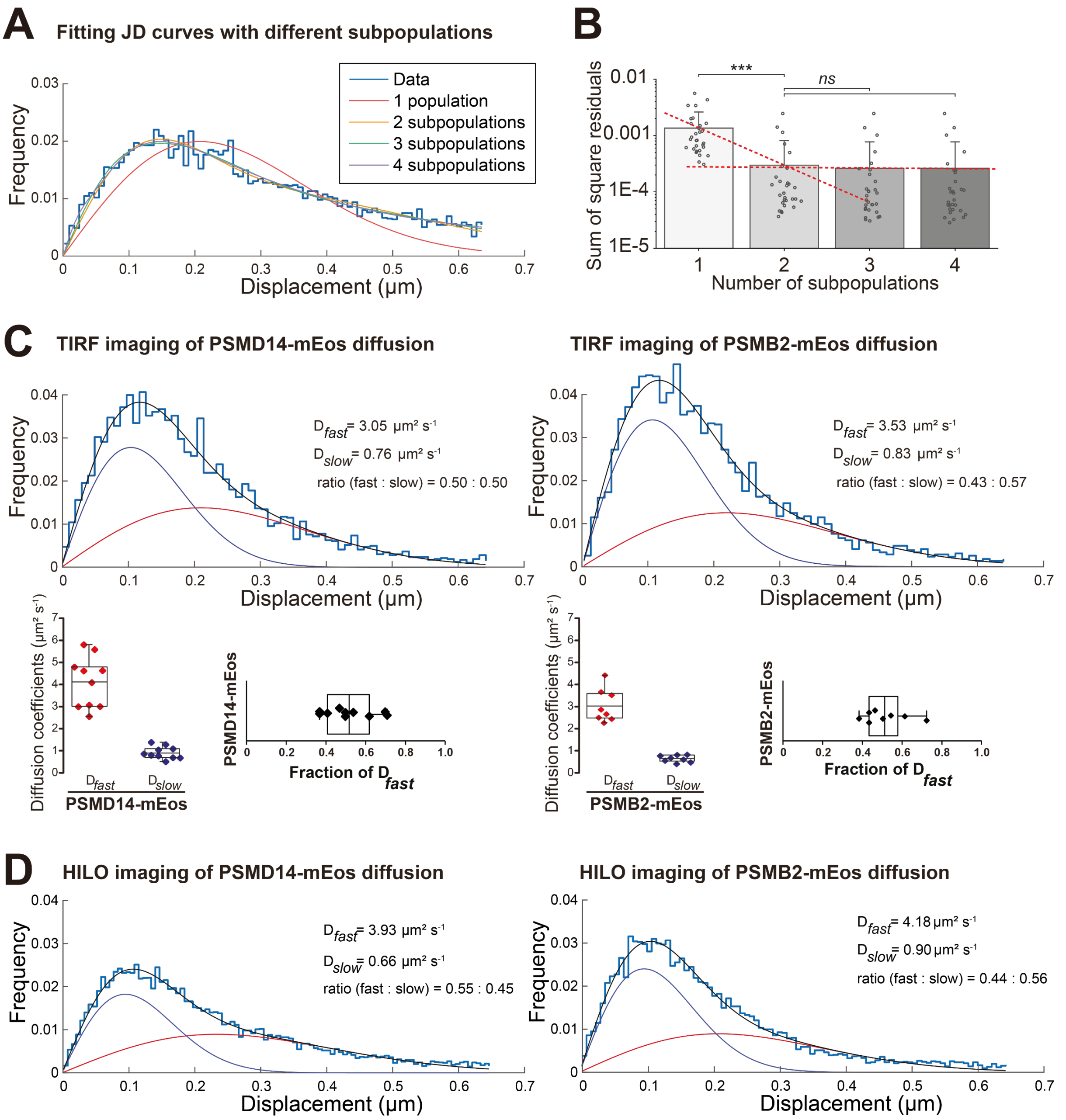

Figure S9. Jump distance frequency distributions are fitted to two sub-populations. (**A**) A frequency distribution plot showing jump distances calculated from one cell expressing PSMD14-eGFP from a SPT experiment. The frequency distribution (blue line) was fitted to non-Gaussian distributions showing one (red line), two (orange line), three (green line) or four (purple line) different subpopulations representing different modes of proteasome diffusion. (**B**) The sum of squared residuals from 30 cells, fitted to four different models with increasing numbers of subpopulations of proteasome diffusion. From these 30 cells, a total of 122 277 tracks were recorded, giving 835 106 jump distances. No further improvement in fitting was observed with models with more than two sub-populations, as shown by statistical tests and the red dotted lines that serve as an elbow test. Scatter points show values for individual cells, the bar represents the mean of all cells, and the error bar is the standard deviation measured across all cells. The elbow test shows an intersection at the model describing two sub-populations, and statistical significance was calculated using Student's *t-test*, where no

significant difference (n.s.) $p>0.05$, * $p\leq0.05$, ** $p\leq0.01$ and *** $p\leq0.001$. (**C**) Single-particle tracking of PSMD14-mEos and PSMB2-mEos cells. (**A**) TIRF imaging of (*left*) PSMD14-mEos cells (mean ± SD for $D_{fast} = 4.1 \pm 1.1$ and $D_{slow} = 0.9 \pm 0.3$ $\mu m^2 s^{-1}$, relative abundance of D_{fast} 0.52 ± 0.1, n = 10 cells) and (*right*) PSMB2 -mEos cells ($D_{fast} = 3.05 \pm 0.8$ and $D_{slow} = 0.7 \pm 0.2$ $\mu m^2 s^{-1}$, where D_{fast} represents 0.51 ± 0.1, n = 8 cells). (**D**) Tracking in HILO mode (n = 13 and n = 16 cells, respectively, of PSMD14-mEos and PSMB2-mEos cells) gives similar diffusion coefficients as in **Figure 4**, suggesting that proteasomal particles diffuse with similar behavior across the whole cell. Results from PSMD14-mEos are similar to PSMD14-eGFP, suggesting that changes in FP do not affect the translocation of the labeled subunit. Due to increased photo-bleaching in HILO mode, tracks from multiple cells were combined for jump distance analysis.

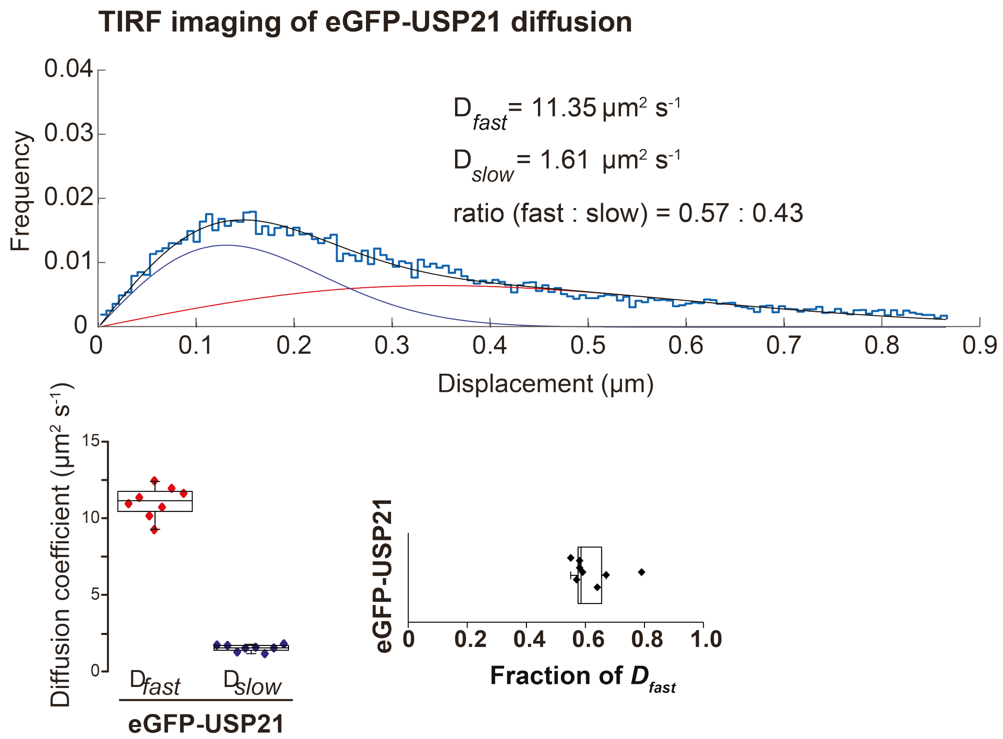

Figure S10. Single-particle tracking of eGFP-USP21 expressed in HEK cells (n = 8) used as a control protein with similar molecular weight as PSMD14, presented as in **Figure 4**. USP21 does not form part of the proteasome.

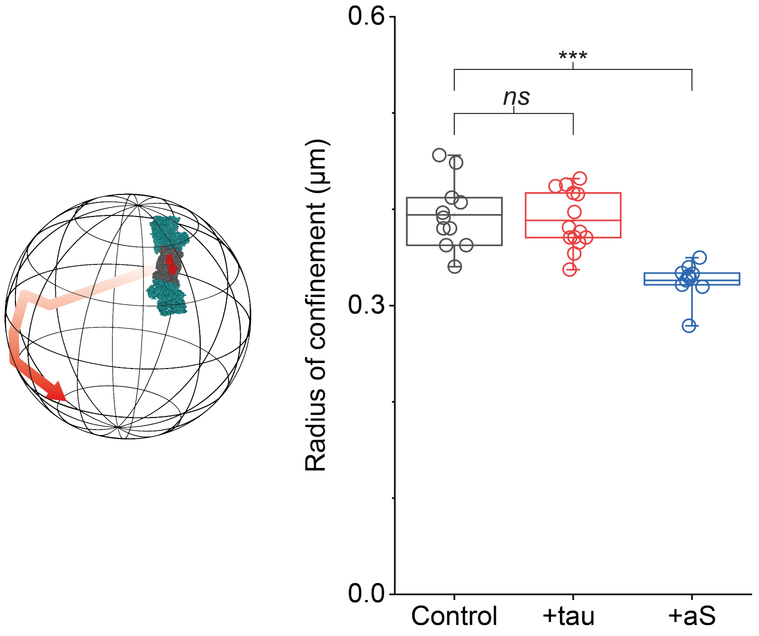

Figure S11. Proteasomes undergoing confined diffusion were identified from cells expressing PSMB2-eGFP during single-particle tracking experiments. MSD curves calculated from single-particle tracks of these proteasomes were fitted to quantify the radius of confinement of cells treated with tau (n = 14 cells and 12 876 confined tracks) and aS (n = 10 and 7 523 confined tracks) aggregates for 12 hrs compared with resting cells (n =11 cells and 14 332 confined tracks).

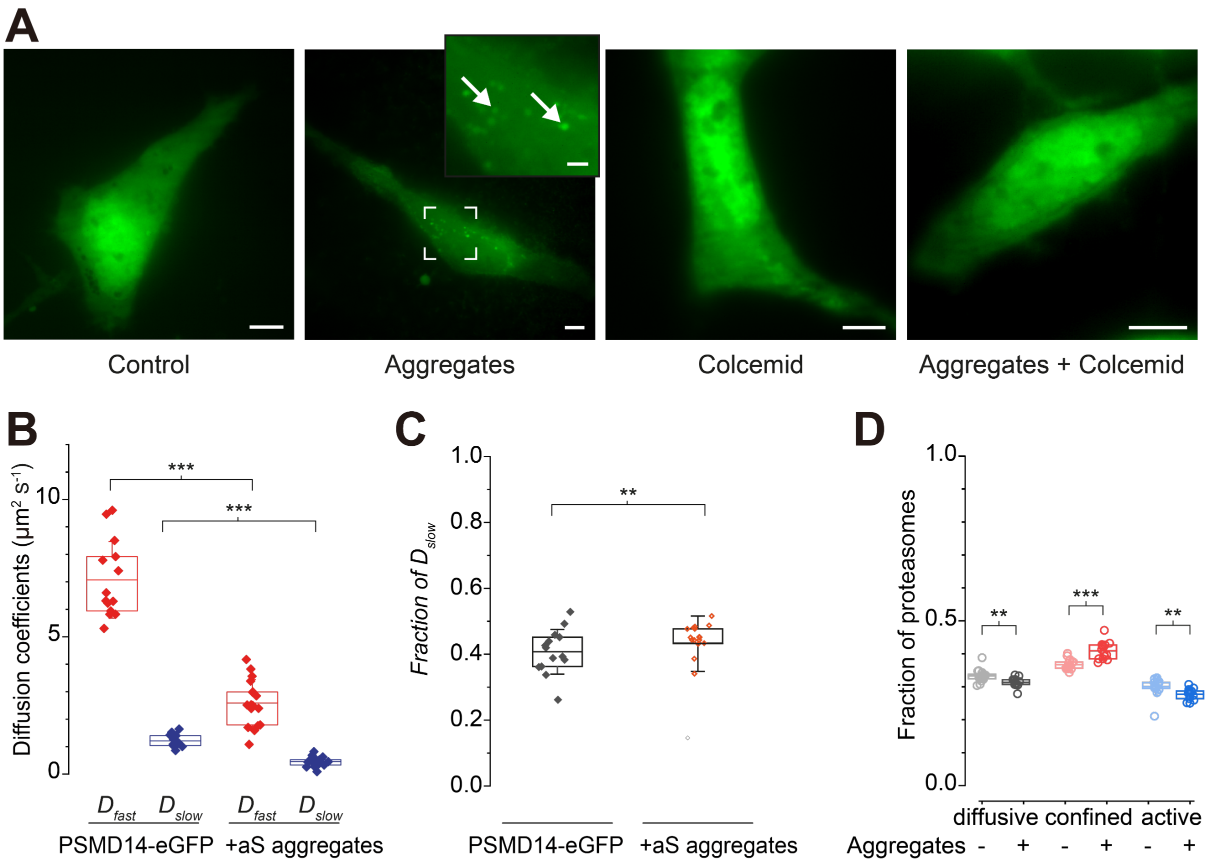

Figure S12. Proteasomes form TAADs in SH-SY5Y cells in response to aS aggregate invasion. (**A**) SH-SY5Y cells expressing PSMB2-eGFP differentiated with retinoic acid and BDNF show diffuse distribution of cells throughout the cell volume. These cells form TAADs in response to incubation with 1 µM aS aggregates for 24 hrs. White arrows in inset point to examples of TAADs. TAADs were not observed when cells were also treated with 0.25 µM Colcemid. Scale bars represent 5 µm, and 2 µm (inset). (**B**) JD analysis from SPT experiments of SH-SY5Y cells showed decreased diffusion coefficients for both D_{fast} and D_{slow} subpopulations, and (**C**) also demonstrated a shift in the fraction of D_{slow} proteasomes within each cell. (**D**) MSD analysis also revealed a significant increase in confined proteasomes within these cells, which was accompanied by a decrease in the fraction of proteasomes undergoing active transport and free diffusion. SPT experiments were conducted with $n_{resting}$ = 17 cells and $n_{aggregate}$ = 14 cells, with a total of 108 231 and 113 198 tracks, respectively. Statistical significance was calculated using Student's *t-test*, where no significant difference (n.s.) $p>0.05$, * $p\leq0.05$, ** $p\leq0.01$ and *** $p\leq0.001$.

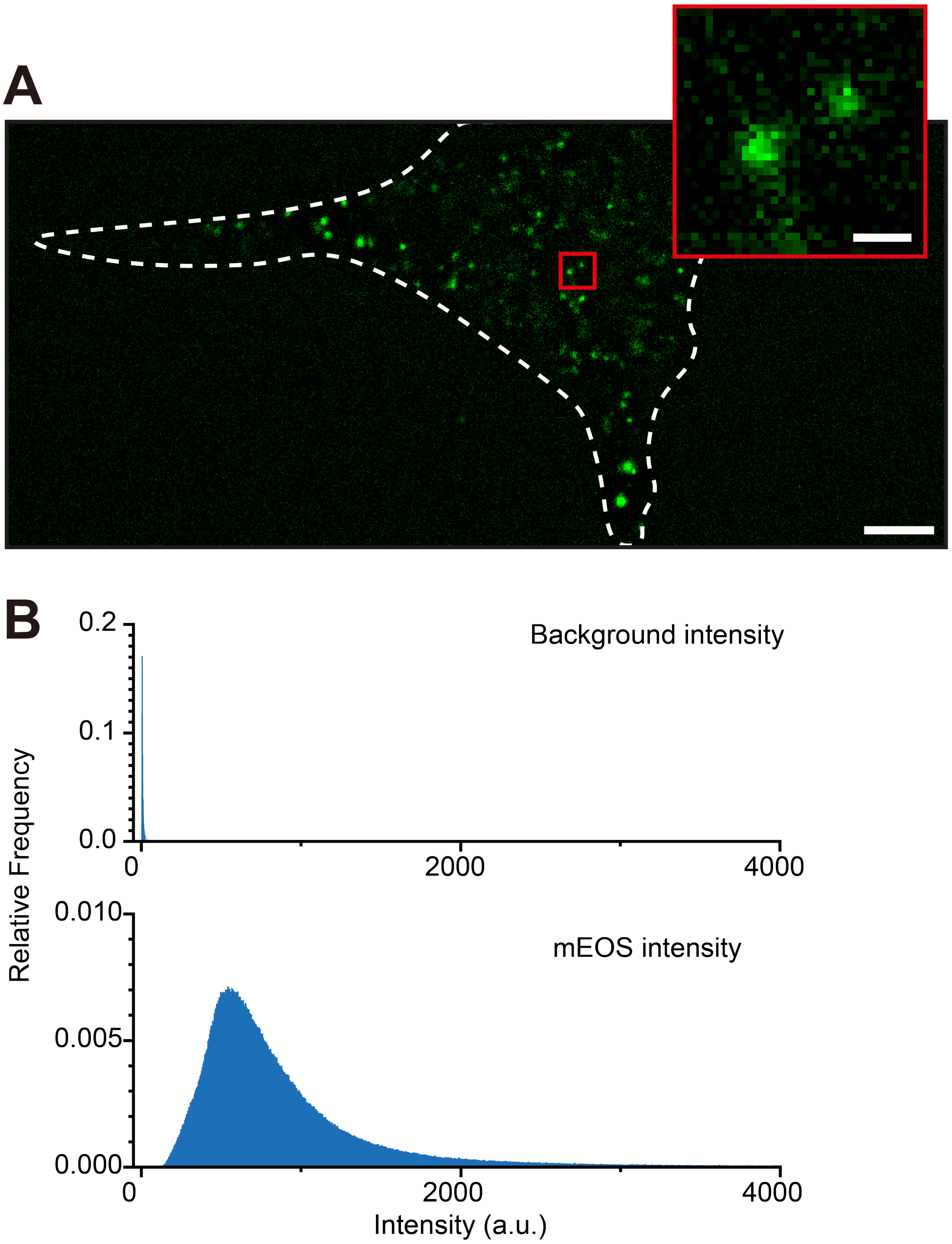

Figure S13. SMLM localizations from individual proteasomes labeled with mEos. **(A)** Diffraction-limited image from a SMLM movie showing individual PSMD14-mEOS particles blinking inside a cell. Inset shows two mEos particles, and the cell border is shown as a white dashed line. Scale bars represent 5 µm, and 500 nm (inset). **(B)** Localizations from 146 cells were fitted to point-spread functions (PSFs). Background intensities were also recorded from pixels surrounding each PSF. The total intensities of the background pixels (top) and the pixels within each PSF (bottom) are plotted from 1 274 188 localizations.
